# Vitellogenin and Vitellogenin-Like Genes in the Brown Planthopper

**DOI:** 10.3389/fphys.2019.01181

**Published:** 2019-09-18

**Authors:** Yan Shen, Yuan-Zhi Chen, Yi-Han Lou, Chuan-Xi Zhang

**Affiliations:** State Key Laboratory of Rice Biology, Ministry of Agriculture Key Lab of Molecular Biology of Crop Pathogens and Insects, Institute of Insect Science, Zhejiang University, Hangzhou, China

**Keywords:** *Nilaparvata lugens*, vitellogenin, vitellogenin-like, nymph, embryogenesis

## Abstract

Vitellogenin (Vg) is precursor of vitellin. Here, we identified a *Vg* (*NlVg*) and two *Vg*-likes (*NlVg*-like1 and *NlVg*-like2) in the brown planthopper, *Nilaparvata lugens*. Phylogenetic analyses showed that NlVg-like1 and NlVg-like2 are not clustered with the conventional insect Vgs associated with vitellogenesis. Temporo-spatial expression analyses showed that the *NlVg* and *NlVg*-like2 transcript levels increased significantly 24 h after emergence and were primarily expressed in female adults. However, *NlVg*-like1 was expressed during all stages, and in both genders. Tissue-specific analyses showed that all three genes were most highly expressed in the fat body. The injection of double-stranded RNA targeting *NlVg* showed that *NlVg* is essential not only for oocyte development but also for nymph development. The knockdown of *NlVg*-like1 in female adults resulted in failure to hatch or death before eggshell emergence in 18% of offspring embryos, suggesting that *NlVg*-like1 plays an important role during late embryogenesis. Approximately 65% of eggs laid by females that were treated with double-stranded RNA targeting *NlVg*-like2 failed to hatch, indicating that *NlVg*-like2 plays a role in nutrition absorption during oocyte, or embryonic development. Our results illustrate the structural and functional differences among the *Vg* and *Vg*-like genes and provide potential targets for RNA-interference-based insect pest management strategies.

## Introduction

The brown planthopper (BPH), *Nilaparvata lugens*, a phloem-feeding hemipteran insect, has emerged as a major pest for rice plants in Asian countries. With 24–33 ovarioles per ovary, the BPH has a robust capacity for producing offspring, and each female adult can produce more than 400 offspring throughout the lifetime of them under appropriate conditions (Chen et al., 1979). The strong fecundity of the BPH represents a primary cause for the serious economic losses associated with the BPH and is regulated by ovary development and environmental conditions. A key process associated with the reproductive success of oviparous species is the biosynthesis of vitellogenin (VTG, Vg) during oogenesis and its accumulation in oocytes (Raikhel, 1992).

Vg belongs to the large lipid transfer protein (LLTP) superfamily, which includes microsomal triglyceride transfer protein (MTP), apolipoprotein (APO), and Vg. This superfamily originates from a series of duplications of *Vg*, and the LTTP superfamily has been hypothesized to be the oldest gene family (Avarre et al., 2007). Several studies have verified the structural and functional relationships among LLTP superfamily members, which have diverse functions. In general, Vgs generally contain a vitellogenin_N domain at the N-terminus, which is also known as the lipoprotein N-terminal domain (LPD_N); a 1943 domain (DUF1943) of unknown function; and a C-terminal von Willebrand factor type D domain (VWD). These domains can be found across an extensive range of proteins (Banaszak et al., 1991; Li et al., 2003; Roth et al., 2013). APOs contain the LPD_N, DUF1081, and VWD domains, whereas MTPs only contain the LPD_N domain. The primary yolk protein precursor family of Vgs (average of 1,300–2,000 amino acids aa long) act as the major energy sources during the embryonic period. This family contains both conventional Vgs and Vg-like protein families, including the immune-related protein family in insects (IP), a protein family of unknown function in fishes (UP), and novel hymenopteran Vg classes (Vg-like A, Vg-like B, and Vg-like C) (Morandin et al., 2014). The APO family (average of 3,200–4,400 aa long, which represent the longest sequence lengths among all LLTPs) includes proteins that are indispensable during the formation and secretion of very low–density lipoproteins, and play important roles during cholesterol and plasma triacylglycerol circulation and metabolism. The MTP family (average, 800–1,000 aa long, which represents the shortest sequence lengths among all LLTPs) transports neutral lipids between membranes *in vitro* and participates in the formation of nascent lipoprotein particles (Wu et al., 2013).

Vgs are large, oligomeric glycolipophosphoproteins, composed of at least 2 subunits (apoproteins), which are synthesized in the fat body (Hagedorn and Kunkel, 1979) as either single, or multiple precursors. Vgs are cleaved proteolytically into the major Vg polypeptide subunits, which are secreted into the hemolymph, where they accumulate via pinocytosis to form vitellin in the developing oocyte (Raikhel and Dhadialla, 1992). Vgs are stored as Vn in crystalline form after incorporation into oocytes and provide amino acids, lipids, fats, carbohydrates, vitamins, microelements, and other nutrients to the maturing oocyte (Tufail and Takeda, 2008). Although the fat body is the exclusive site of Vg synthesis in most insects (Valle, 1993), Vgs are also produced by the ovarian follicular epithelium in some Coleoptera, and Diptera species (Belles, 1998). More surprisingly, in some species, Vg is present not only in females but also in males (Trenczek and Engels, 1986; Piulachs et al., 2003).

The conserved structural domains of Vgs give rise to multiple functions. In addition to the primary function of egg yolk formation, Vg is associated with other functions, including the sexual division of foraging, the specialization of labor, and pleiotropic effects in the female castes of social Hymenoptera. Vg has also been associated with the regulation of hormonal dynamics, longevity, and gustatory responsiveness (Engels, 1974; Amdam and Omholt, 2003; Guidugli et al., 2005; Nelson et al., 2007; Munch and Amdam, 2010). Moreover, Vg can protect some species from oxidative stress, such as the honey bee, nematodes (*Caenorhabditis elegans*), and fish (*Anguilla japonica*) (Ando and Yanagida, 1999; Nakamura et al., 1999; Seehuus et al., 2006). In addition, Vg plays important roles during immunity in amphioxus (Zhang et al., 2005), fish (Li et al., 2008, 2009; Liu et al., 2009; Garcia et al., 2010), bay scallop (Wu et al., 2015), mud crab (Yang et al., 2016), and noble scallop (Zhang et al., 2016).

To date, Vg has been studied extensively in numerous species, including both vertebrates and invertebrates, and has been cloned from seven insect orders: Blattodea, Orthoptera, Coleoptera, Diptera, Lepidoptera, Hemiptera, and Hymenoptera. *Vg* copy numbers are highly variable among different species, ranging from one to several (Morandin et al., 2014). Specifically, the mosquito (*Aedes aegypti*), and the ant (*Linepithema humile*) (Corona et al., 2013) both have five copies (Romans et al., 1995), which are the largest number of *Vg* genes among insect species, whereas the honeybee (*Apis mellifera*) (Piulachs et al., 2003; Amdam et al., 2012), the ant (C*amponotus floridanus*) (Corona et al., 2013), and the silkworm (*Bombyx mori*) (Yano et al., 1994) have only a single copy, which is the lowest copy number. These copy number differences have been attributed to gene duplication events, which are thought to be a crucial source of evolutionary novelties that result in new gene expression patterns and functions (Lynch and Conery, 2000), indicating that copy number variations for *Vg* genes may be associated with insect adaptation and evolution (Lynch and Force, 2000; Garcia et al., 2010). More importantly, ancient duplications may result in different physiological effects among different species (Innan and Kondrashov, 2010). For example, the white perch (*Morone americana*) has three types of Vg proteins: VtgAa, VtgAb, and VtgC, which have different gene expression patterns in the ovary, plasma, and liver during pre-, early-, mid-, and post-vitellogenic oocyte growth, and appear to be associated with different functions. The relative levels between VtgAa and VtgAb are associated with egg buoyancy (determining whether eggs are neutrally buoyant or demersal adhesive), and the VtgC protein content may affect nutrition supplementation during early embryonic development (Schilling et al., 2015).

Due to the various gene duplication events, cleavage sites, and temporo-spatial expression patterns of Vg proteins, a new and diverse class of *Vg* genes has emerged in several insects over time, termed the *Vg*-like genes. For example, three *Vg* homologs, *Vg*-likeA, *Vg*-likeB, and *Vg*-likeC, which are orthologous to the conventional *Vg*s, have emerged in Hymenoptera (Morandin et al., 2014). The conserved protein domains in these genes vary and are unlikely to be associated with vitellogenesis. In particular, Vg-likeA plays an important role in determining longevity of winter bees. Vg-likeB is linked to the oxidative stress response. The function of Vg-likeC remains generally unknown, although it does not appear to be associated with anti-aging functions (Salmela et al., 2016). In the potato psyllid, *Bactericera cockerelli, BcVg1*-like encodes a conventional Vg, whereas *BcVg6*-like appears to be associated with lipid movement (a lipid transporter during metamorphosis), protection from reactive oxygen species, and immune defense (Ibanez et al., 2017, 2018). There was only one *Vg*-like gene in the bed bug, *Cimex lectularius*, and it may have acquired a novel function that differs from that of *ClVg*; however, the function of the *ClVg*-like gene remains unclear and requires further study (Moriyama et al., 2016).

*Vg* is not only expressed in female adults but can also be detected in other stages in many insect species, and many insects possess several *Vg*-like genes. However, the biological functions of *Vg* in nymphs and the functions of *Vg*-like genes in all stages remain largely unclear. In this study, we identified three *Vg* or *Vg*-like genes in *N. lugens*. To better understand the evolution and functions of the *Vg* and *Vg*-like genes in this phloem-feeding, destructive rice pest, the complete open reading frame (ORF) sequences of *NlVg, NlVg*-like1, and *NlVg*-like2 from *N. lugens* were cloned, analyzed, and compared with known insect *Vg*s, *Vg*-like genes, and other LLTP family sequences to investigate their phylogenetic relationships. Then, the three genes were characterized functionally to enable a better understanding of the roles played by these novel *Vg*s in the phloem-feeding species and to determine whether these genes represent potential targets for BPH management based on RNA interference (RNAi) technologies.

## Materials and Methods

### Insects

The *N. lugens* colony used for this study was obtained from local rice fields on the Huajiachi Campus of Zhejiang University, Hangzhou, China. The colony was maintained on fresh rice seedlings at 26 ± 0.5°C, with 50 ± 5% relative humidity, under a 16 h light:8 h dark photoperiod. Because ovary development is delayed in the long-winged morphs of many insects, including *N. lugens*, and short-winged morphs often have higher fecundity than long-winged morphs (Zera and Denno, 1997), short-winged BPHs were used in this study, unless otherwise specified.

### Identification and Analyses of *Vg* and *Vg*-like Genes

The cDNA sequences of *Vg* and the *Vg*-like genes were obtained from transcriptome databases and used as query sequences against *N. lugens* genome databases (GenBank accession number AOSB00000000) using the National Center for Biotechnology Information (NCBI)-basic local alignment search tool (BLAST)-2.5.01 (using BLASTn, default parameters) to obtain the genome sequences.

The exon-intron organization of *Vg* and *Vg*-like genes were predicted using Splign (https://www.ncbi.nlm.nih.gov/sutils/splign/splign.cgi). The domain structures of Vg and Vg-like proteins were predicted using the Simple Modular Architectural Research Tool (SMART; http://smart.embl-heidelberg.de/) and a specialized BLAST tool for identifying conserved domains within sequences that can be found on the NCBI website (https://www.ncbi.nlm.nih.gov/Structure/cdd/wrpsb.cgi).

To investigate the evolutionary relationships among the *Vg* and *Vg*-like genes identified in the present study, the gene sequences were used to search for homologous genes in the NCBI non-redundant database (https://blast.ncbi.nlm.nih.gov/Blast.cgi). Phylogenetic analyses were performed using the deduced amino acid sequences of the identified Vg and Vg-like proteins and Crustacea Vgs and Crustacea APOs that were selected from different representative species among 15 orders. Multiple sequence alignments were performed using CLUSTALX (Larkin et al., 2007). Phylogenetic trees were constructed using MEGA7 (Kumar et al., 2016), using the maximum likelihood method and bootstraps with 1,000 replications.

### Isolation of Total RNA and Reverse Transcription-PCR

Total RNA was isolated from whole insects at various developmental stages or from tissue samples, using a TRIzol Total RNA Isolation Kit (TaKaRa, Dalian, China) according to the manufacturer's protocol. Developmental samples were collected from eggs (every 24 h after the eggs were laid, *n* = 100), first-instar nymphs (every 24 h after molting, *n* = 80), second-instar nymphs (every 12 h after molting, *n* = 60), third-instar nymphs (every 12 h after molting, *n* = 40), fourth-instar nymphs (every 12 h after molting, *n* = 20), fifth-instar nymphs (every 12 h after molting, *n* = 15), female adults (every 12 h after molting, *n* = 15), and male adults (every 12 h after molting, n = 15). Similarly, tissue samples, including the abdominal integument (n = 50), gut (*n* = 50), fat body (*n* = 30), and ovaries (*n* = 30) were dissected from females 48–72 h after emergence; the testes (*n* = 40) were dissected from males 48–72 h after emergence.

The concentration of total RNA was measured using a NanoDrop 2,000/2,000 c spectrophotometer (Thermo Fisher Scientific, Bremen, Germany), and 1,000 ng RNA was used for reverse transcription (RT) in a 10-μL reaction with a ReverTra Ace qPCR RT Master Mix and genomic DNA (gDNA) Remover Kit (ToYoBo, Osaka, Japan), according to the manufacturer's recommendations. The synthesized cDNAs were used as templates to amplify the *NlVg, NlVg-*like1, and *NlVg*-like2 coding sequences, using Phanta Max Super-Fidelity DNA Polymerase (Vazyme, Jiangsu, China) according to the manufacturer's protocol. The PCR procedure was as follows: pre-denaturation at 95°C for 3 min, 35 cycles of 95°C for 15 s, 55°C for 15 s, and 72°C for 15 s, and an additional extension at 72°C for 5 min. The PCR products were cloned into a pEASY–Blunt Cloning Vector (TransGen, Beijing, China) and sequenced. Table S1 lists the primers used during the PCR amplifications.

### Real-Time qPCR

To investigate the developmental and tissue-specific expression patterns of *NlVg, NlVg*-like1, and *NlVg*-like2, real-time qPCR was conducted using gene-specific primer pairs designed by Primer Premier 5 (Table S1) and the cDNA was prepared as described above. Each 20-μL reaction contained 2 μL 10-fold diluted cDNA, 0.6 μM each primer, and 10 μL SYBR Premix Ex Taq (Bio-Rad, Hercules, CA, USA), and was conducted on a CFX96 Real-Time PCR Detection System (Bio-Rad) under the following conditions: initial denaturation at 95°C for 30 s, followed by 40 cycles at 95°C for 5 s and 60°C for 30 s. The relative RT-qPCR data were analyzed with CFX Manager 3.1 (Bio-Rad). The *N. lugens* 18S rRNA housekeeping gene (*Nl18S*) (GenBank accession number JN662398.1) was used as the reference gene. All RT-qPCR data are presented as relative mRNA expression levels; RT-qPCR was performed for each gene based on independent RNA sample preparation, and consisted of three technical replicates and three biological replicates. The relative quantitative method [2^−ΔΔCt^: 2^−(Cttarget−Ct*Nl18S*)timex−(Cttarget−Ct*Nl18S*)time0^], where timex is any time point and time 0 represents the 1 × expression of the target gene normalized to *Nl18S*; the Ct (cycle threshold) (Schmittgen and Livak, 2008) was used to evaluate the quantitative variation.

### dsRNA Preparation

To minimize non-target silencing, a unique region of each *NlVg* or *NlVg-*like gene was chosen for the dsRNA design. The coding sequences of *NlVg, NlVg*-like1, *NlVg*-like2, and *GFP* (control) were cloned into the pMD-19T vector (TaKaRa). PCR-generated DNA templates were then used to synthesize the dsRNA, which contained *T7* promoter sequences at each end. Table S1 shows the primers used for dsRNA synthesis. We used a MEGAscript T7 Transcription Kit (Ambion, Austin, TX, USA) according to the manufacturer's instructions to produce the specific dsRNA of each gene. The PCR was performed using Green Taq Mix (Vazyme) in 50-μL reactions, and was as follows: pre-denaturation at 94°C for 5 min, followed by 35 cycles of 94°C for 30 s, 58°C for 30 s, and 72°C for 1 min, and additional extension at 72°C for 7 min. Subsequently, dsRNA was purified using LiCl precipitation, resuspended using nuclease-free water, and quantified spectrophotometrically using a NanoDrop 2,000/2,000 c spectrophotometer (Thermo Fisher Scientific, Bremen, Germany). The dsRNA products' quality and size were verified by 2% agarose gel electrophoresis.

### Effects of RNAi on *N. lugens*

RNAi was performed as described previously (Liu et al., 2010; Xu et al., 2015). Briefly, newly emerged adult female BPHs (within 2 h) were used for microinjections. First, the female BPHs were anesthetized with carbon dioxide for 15–20 s. Then, ~50 ng dsRNA was injected into each BPH using a FemtoJet (Eppendorf-Netheler-Hinz, Hamburg, Germany). Post-injection, the female BPHs were placed on 4–5-leaf–stage rice seedlings for 5 h, and then an equal or greater number of male BPHs were placed in the same jar. Fifty injected adult female BPHs were used for each treatment, with three replicates performed.

The efficiency of RNA silencing was evaluated by real-time quantitative PCR (RT-qPCR), 72 h after injection. The female BPHs from day 4 to day 9 after emergence were dissected. Approximately 20 females per treatment were maintained and counted daily until day 8 after injection. For dissection, the insects were anesthetized on ice and placed in a Petri dish, and then the cuticle was carefully removed, and the tissues were removed using forceps and placed in a drop of phosphate-buffered saline (PBS: 0.9% NaCl, 0.02% KCl, 10 mM Na_2_HPO_4_, 2 mM KH_2_PO_4_, [pH 7.4]). Images of the insects and of their internal reproductive systems were captured with a DFC320 digital camera (Leica, Germany) attached to a Leica S8 APO stereomicroscope, using the LAS V3.8 digital imaging system (Leica, Germany).

### Western Blotting

BPH nymphs of all stages (0–12 h first, second, third, four, **fifth** instar) and 3-day-old females, as well as second- and fifth-instar nymphs and adult females that had been treated with ds*NlVg* and ds*GFP* in their early stages (0–12 h) were homogenized in radioimmunoprecipitation assay (RIPA) buffer (Thermo Fisher Scientific), quantified using the bicinchoninic acid (BCA) method (catalog no. 23227, Thermo Fisher Scientific), and electrophoresed via sodium dodecyl sulfate–polyacrylamide gel electrophoresis (SDS-PAGE). The optimized western blotting protocol consisted of the following steps: First, the polyvinylidene fluoride (PVDF) membrane (Merck KGaA, Darmstadt, Germany) was activated with methanol for 30 s. Beginning from the bottom up with a foam pad, the transfer box was assembled using filter paper, gel, membrane, another layer of filter paper, and foam pad. Then, protein samples were transferred to the PVDF membrane in buffer (2.8 g Tris base, 2.9 g glycine, 200 mL methanol in 800 mL ddH_2_O [double-distilled water]) (Thermo Fisher Scientific) at 60 V for 2 h 20 min. Next, the PVDF membrane was blocked with blocking buffer (5% skim milk powder in 1 × TTBS; 1 × TTBS; 10 × TBS [Tris-buffered saline] with 1% Tween 20) at room temperature for 1 h 30 min. The primary antibodies against NlVg and β-actin (1:10,000)prepared in our laboratory (Xu et al., 2015; Zhuo et al., 2018) were then added to the blocking buffer at room temperature for 2 h 30 min or at 4°C overnight. The PVDF membrane was washed with 1 × TTBS **three** times for 15 min each. Then, the secondary antibody (goat anti-mouse; HuaBio, Hangzhou, China) was added with fresh blocking buffer (0.5% skim milk powder in 1 × TTBS) at room temperature for 2 h 30 min. The PVDF membrane was washed with 1 × TBS three times for 10 min each. The chemiluminescence apparatus (Bio-Rad) was prepared 20 min in advance, and the PVDF membrane was visualized with color-developing agent (Bio-Rad); a standard protein ruler was used (catalog no. 26616, Thermo Fisher Scientific). Samples were collected from 0 to 12 h first-instar nymphs (*n* = 80), second-instar nymphs (*n* = 60), third-instar nymphs (*n* = 40), fourth-instar nymphs (*n* = 20), fifth-instar nymphs (*n* = 15), and 3 day-old females (*n* = 15). Similarly, samples treated with *dsNlVg* and *dsGFP* in their early stages (0–12 h): second-instar nymphs (*n* = 60), and fifth-instar nymphs (*n* = 15), and adult females (*n* = 15).

### Triglyceride Assay

The triglyceride content of the BPHs was measured at 550 nm using a triglyceride assay kit (Applygen Technologies, Beijing, China). Total protein concentration was estimated using the BCA method (catalog no. 23227, Thermo Fisher Scientific) according to the manufacturer's instructions and is expressed as μmol triglyceride per μg total protein.

### Statistical Analysis

Data are presented as mean ± SEM from three independent biological replicates, u*Nl*ess otherwise noted. Statistical analysis was performed using GRAPHPAD PRISM 7.0 (GraphPad Software, La Jolla, CA, USA). Differences between two groups were compared using a two-tailed unpaired *t*-test (Tukey's multiple comparisons test for multigroups) at the significance levels of ^*^P < 0.05, ^**^P < 0.01, ^***^P < 0.001. One-way analysis of variance (ANOVA) was applied for comparing the differences among more than two samples.

## Results

### Sequencing and Phylogenetic Analysis

**Figure 1:**
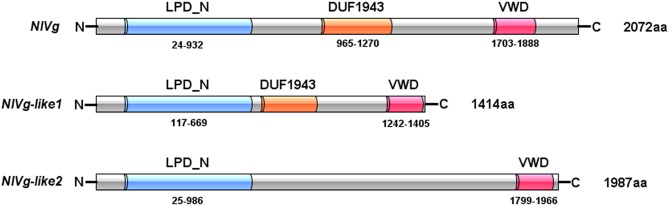
Domain architecture of NlVg, and NlVg*-*like1, and NlVg*-*like2. All of these proteins contain the LPD_N and VWD domains, whereas only NlVg*-*like2 has no predicted DUF1943.

Three Vg homologs were identified from the BPH transcriptome (Xue et al., 2010) and genome (Xue et al., 2014), using the Vg amino acid sequences from *A. mellifera, Ae. aegypti*, and *Acyrthosiphon pisum* as queries, and were tentatively named NlVg (2,072 aa), NlVg-like1 (1,414 aa), and NlVg-like2 (1,987 aa) (Figure 1, Figures S1–S3). The coding sequences of the three genes were validated through cloning and sequencing (GenBank accession numbers: AB353856, MK779307, and MK779308). Comparisons among the transcriptome and genome sequences of the three genes revealed that *NlVg, NlVg*-like1, and *NlVg*-like2 contain 25, 22, and 18 exons, respectively (Figure S4). All three putative proteins contain the LPD_N and VWD domains, two large domains that are usually found in conventional Vgs and other LLTPs (Wu et al., 2013). In addition, NlVg and NlVg-like1 contain the unknown function motif 1943 (DUF1943) (Figure 1), which is also found in conventional Vgs from other insect species.

**Figure 2:**
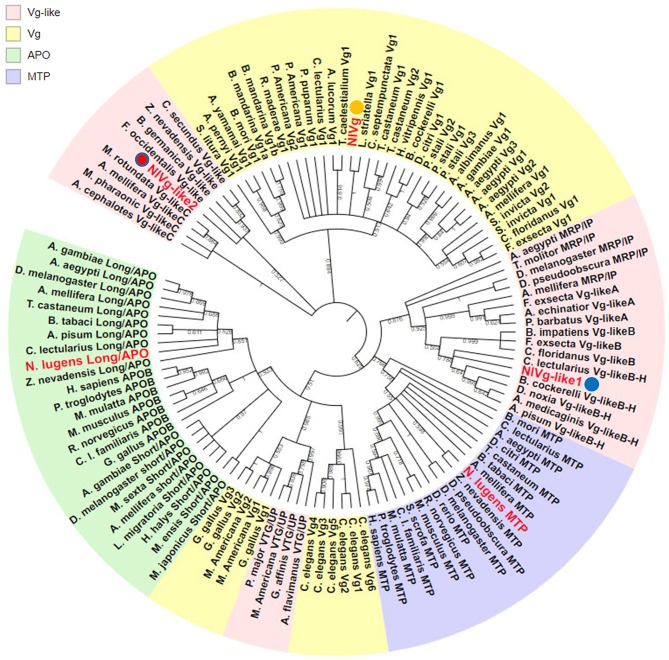
Phylogenetic relationship of *NlVg, NlVg-*like1, and *NlVg-*like2 with members of the LLTP superfamily. Phylogenetic analysis of NlVg and NlVg-like proteins involved other members of the LLTP superfamily, including conventional Vgs, APO family, MTP family, Vg-like A, Vg-like B, Vg-like C, IP, and UP. Cladograms show bootstrap values; only branches with ≥50% support are shown. The best-fit amino acid sequences of 119 proteins from 14 different orders in five classes were chosen for alignment and analysis. Numbers at the nodes denote posterior probabilities (support values > 50% are shown). Table S2 lists the detailed information of each protein sequence used.

Using the maximum likelihood method, NlVg, NlVg-like1, and NlVg-like2 were compared phylogenetically with conventional Vgs, the APO family, the MTP family, three new hymenopteran Vg classes (Vg-likeA, Vg-likeB, Vg-likeC), IP, and UP (Figure 2). NlVg appeared to belong to the conventional insect Vgs, grouping with hemipterans, whereas NlVg-like1 and NlVg-like2 were divided into different groups. Specifically, NlVg-like1 was closely related to *B. cockerelli* Vg-likeB and clustered with a described group termed Hemiptera Vg-likeB (Morandin et al., 2014; Ibanez et al., 2018), which is supposed to be a sister clade of hymenopteran Vg-likeB. Interestingly, *NlVg*-like2 was located with other *Vg*-like genes belonging to hemimetaboly and was placed within the clade composed of Vg-like proteins from western flower thrip (*Frankliniella occidentalis*), German cockroach (*Blattella germanica*), and two termite species (*Zootermopsis nevadensis* and *Cleaveius secundus*). Hence, these three genes diverged from the common ancestor, implying that they may have different functions.

### Temporospatial Expression Patterns

**Figure 3:**
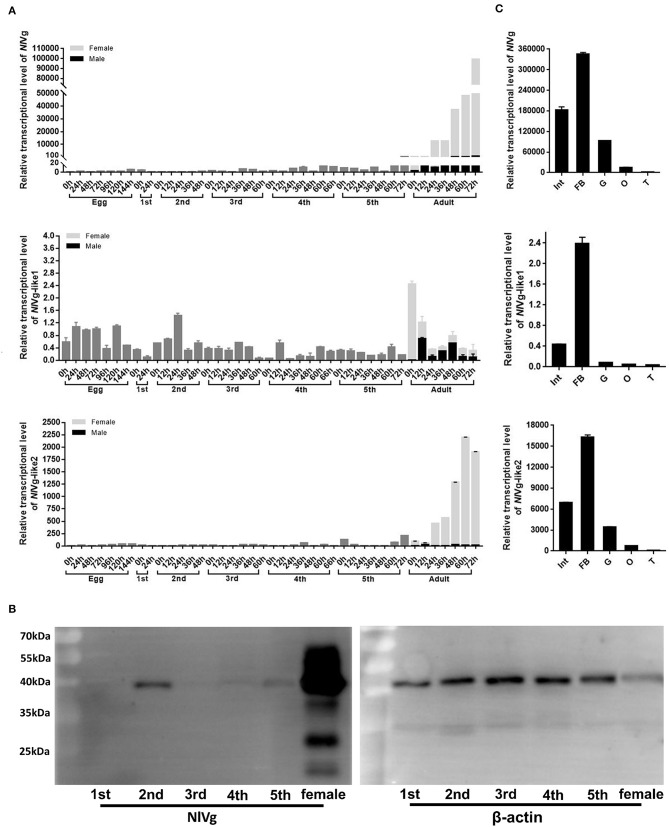
Temporospatial expression patterns of *NlVg, NlVg-*like1, and *NlVg-*like2. **(A)** Temporal expression pattern of the three genes. Samples were collected from different developmental stages of eggs, nymphs (first, second, third, fourth, fifth instar), and adults (female and male). *Nl18S* was used as the internal control gene. The x-axis represents the time points at which insects were collected; the y-axis represents the relative transcript level of the target gene. Results are the mean ± SEM of three biological replicates with their respective three technical replications. **(B)** Western blotting of NlVg in nymphs (first, second, third, fourth, fifth instar), and females. Approximately 8 μg protein from each nymph sample and 4 μg protein from each female adult sample was used. β-Actin was used as the positive control. **(C)** Spatial expression patterns of the three genes in BPH tissues. Total RNA was extracted from the following tissues: abdominal integument (Int, *n* = 50), fat body (FB, *n* = 30), gut (G, *n* = 50), and ovary (O, *n* = 30), which were dissected from the females 48–120 h after emergence; testes (T, *n* = 40) were dissected from the males. *Nl18S* was used as the internal control gene. Data are the relative mRNA levels of *NlVg, NlVg-*like1, and *NlVg-*like2. Results are the mean ± SEM of three biological replicates with their respective three technical replications. Different letters indicate statistical differences by the HSD-test at the 5% level of significance in each histogram.

RT-qPCR results showed that the transcription levels of both *NlVg* and *NlVg*-like2 increased significantly 24 h after emergence and remained at high levels throughout the adult stage. The two genes were expressed almost exclusively in females and were expressed at low or nearly undetectable levels in eggs and nymphs. As ovarian maturation generally occurs 3 days after eclosion, the expression patterns of *NlVg* and *NlVg*-like2 were consistent with oocyte development (Figure 3A). Western blotting also detected high levels of NlVg expression in females. Surprisingly, NlVg could also be detected in nymphs (Figure 3B). Contrary to *NlVg*-like2 and *NlVg*, which were only detected in females, *NlVg*-like1 transcripts were detected in both males and females, with high transcript levels that peaked at ~0 h in female adults. However, female adults had higher levels of *NlVg*-like1 mRNA than males (Figure 3A).

The tissue-specific expression profiles of these three genes were analyzed by RT-qPCR in five tissues (integument, gut, fat body, ovaries and testes) (Figure 3C). Different levels of transcripts for the three genes were detected in almost all the tissues; transcription levels for all three genes were highest in the fat body, followed by those in the integument, gut, and the two reproductive organs.

### Effects of *NlVg, NlVg*-like1, and *NlVg*-like2 on Reproductivity and Embryogenesis

**Figure 4:**
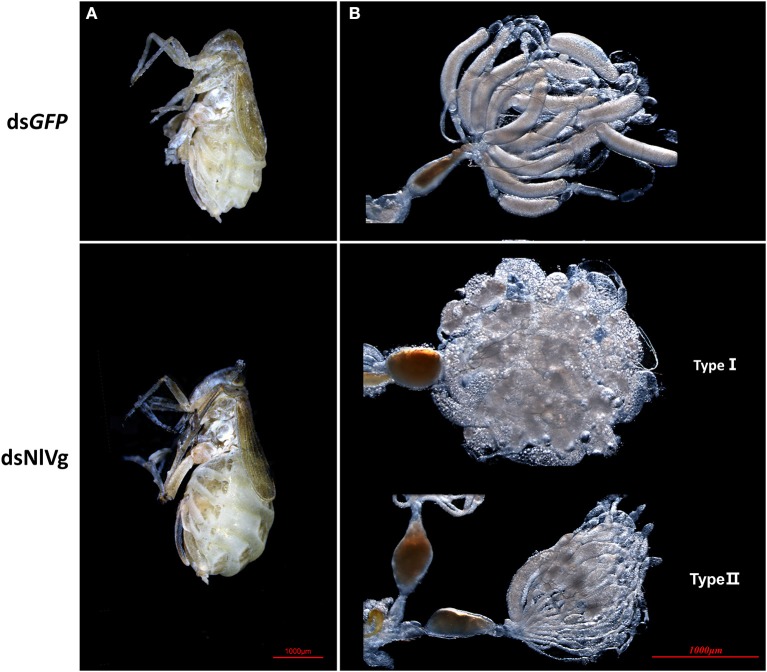
Knockdown of *NlVg* leads to oocyte malformation. **(A)** Female BPHs at day 7 after emergence (*n* = 120). ds*NlVg*-treated BPHs (*n* = 120) had larger abdomens compared to the control, with greatly stretched intersegmental membranes in the abdomen. **(B)** Oocytes derived from ds*GFP* and ds*NlVg-*treated female adults. At 9 days after emergence, oocytes in the ds*NlVg*-treated group were ball-shaped (type I) or filiform (type II) compared with the replete banana-shaped oocytes in the ds*GFP*-treated group.

Based on the developmental expression patterns of *NlVg, NlVg-*like1, and *NlVg-*like2, RNAi experiments were conducted on newly emerged (0–2 h) females before ovary maturation to observe the phenotypes regarding oviposition and the embryonic development of the next generation. *NlVg*-like1, *NlVg*-like2, and *NlVg* mRNA levels were measured at 72 h post-injection, and RNAi efficiently downregulated their expression levels by 82.8, 98.8, and 95.8%, respectively (Figure S5A). The injection of *NlVg* double-stranded RNA (dsRNA) led to remarkable phenotypes. The body sizes of female adults injected with *NlVg* dsRNAs (ds*NlVg*) became substantially larger at day 7 than those of females treated with dsRNAs for green fluorescent protein (ds*GFP*, control), with the obvious stretching of the lateral and intersegmental membranes of the abdomen. However, no obvious external morphological changes were detected in female adults that were treated with dsRNA against *NlVg*-like1 or *NlVg*-like 2 (ds*NlVg*-like1 or ds*NlVg*-like2, respectively; Figure 4A). Subsequently, we found that the ds*NlVg*-treated females were full of hypertrophied fat bodies that stuck to the in walls of the swollen abdomens, in contrast to the thinner abdomens containing smaller fat bodies observed for females in the other two experimental groups and the control group.

**Figure 5:**
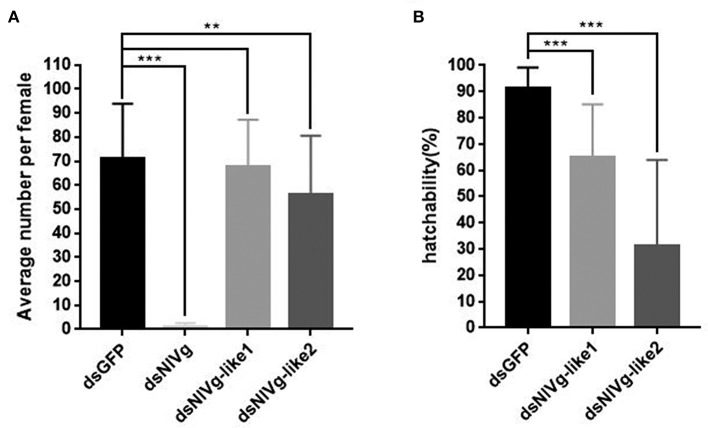
The fecundity (amount of eggs) per female after injection of ds*NlVg*, ds*NlVg-*like1, ds*NlVg-*like2, or ds*GFP* (*n* = 34). Three days after injection, females were paired with male BPHs of the same age and allowed to lay eggs for 3 days. **(A)** Average number of eggs per female. Eggs with eyespots after 4 days were identified as developed eggs. **(B)** Hatchability (%) shows the percentage of hatchlings in fertilized eggs. Results are the mean ± SEM of 34 independent experiments. ^**^P < 0.01, ^***^P < 0.001 (Tukey's multiple comparisons test).

**Figure 6:**
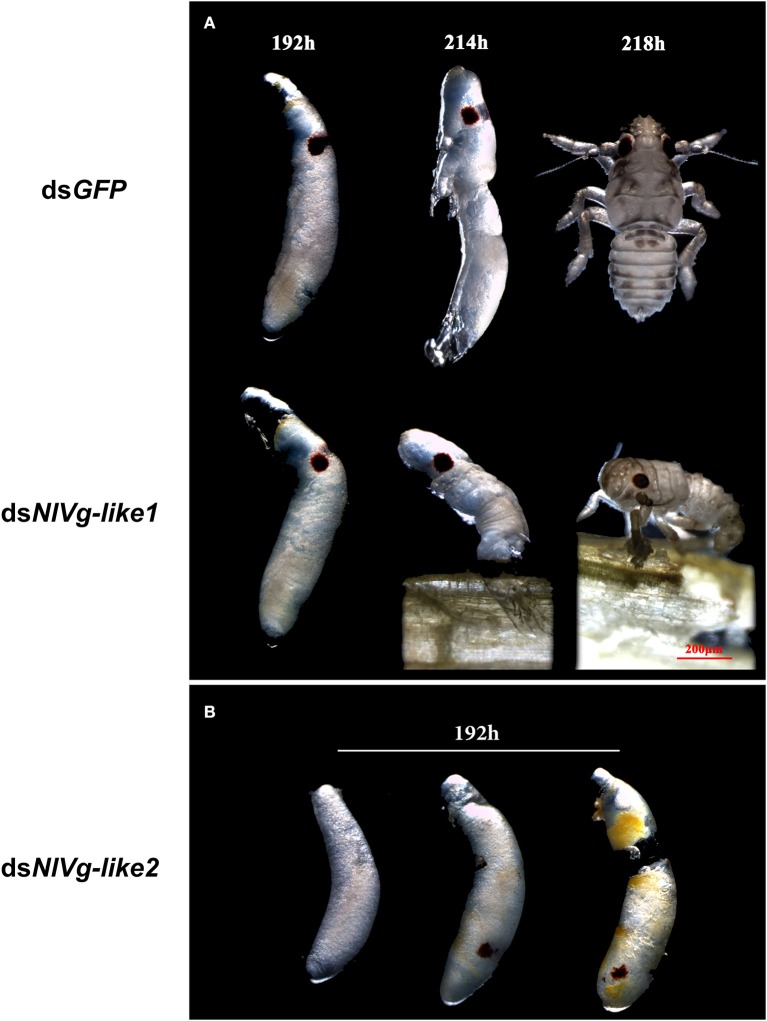
Embryonic development after injection of ds*NlVg-*like1, ds*NlVg-*like2, or ds*GFP* (observed every 24 h after the eggs were laid, *n* = 100). Most first-instar nymphs hatched from the shells at 214–218 h after egg formation. **(A)** Phenotypes of ds*NlVg-*like1 group (*n* = 2,000): 18% of hatchlings failed to exit the egg after 218 h and died before leaving the eggshell, compared to only 2% of individuals in the control that failed the hatching process. **(B)** Phenotypes of abnormal eggs from the ds*NlVg-*like2 group (*n* = 300). Malformed eggs arrested at 0–96 h before eyespot formation or embryo eversion, with abnormal development of most organs. Results are the mean ± SEM of 34 independent experiments.

To observe ovary development, we dissected the ovaries of virgin female adults at day 9 after dsRNA injection. No obvious oocyte malformations were observed in females that had been treated with ds*NlVg*-like1 and ds*NlVg*-like2; every ovariole contained one or two matured and replete banana-shaped oocytes. However, *NlVg* knockdown severely inhibited oocyte growth in the ovarioles, and the oocytes were misshapen, with irregular edges (ball-shaped or filiform), instead of banana-shaped, with smooth chorion (Figure 4B). This malformation indicated that *NlVg* is required for oogenesis and oocyte maturation, and nearly all ds*NlVg*-treated females failed to produce offspring (Figure 5A). Females that were treated with ds*NlVg*-like1 could oviposit normally after mating with wild-type males; however, only 82% of the laid eggs successfully hatched, compared with the 98% hatchability observed for the ds*GFP*-treated group (Figure 6A, Video S1). Females that were treated with ds*NlVg*-like2 also laid eggs normally, but the eggs had significantly decreased hatchability compared with the control group. In addition to only 31.11% of eggs hatching successfully (Figure 5B), the malformed phenotypes of the abnormal eggs varied greatly. Some embryos showed no eyespots; in other eggs, the eyespots appeared near the posterior poles of the eggs, suggesting that blastokinesis did not successfully complete (Figure 6B).

Because *NlVg*-like1 was highly expressed in males, fifth-instar male BPHs (within 2 h after molting) were injected with ds*NlVg*-like1 to determine its functions in males. No obvious malformations were observed. When wild-type females mated with males that were treated with *dsNlVg*-like1, no obvious differences were observed for either fecundity or hatchability.

### Effects of *NlVg, NlVg*-like1, and *NlVg*-like2 on Nymphal Development

**Figure 7:**
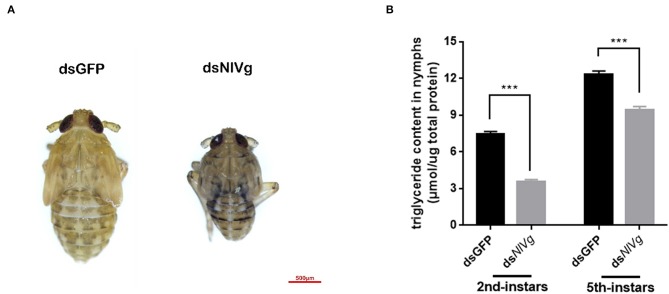
Effects of *NlVg* on nymphal development. **(A)** Injection of ds*NlVg* in nymphs (*n* = 100) caused a lethal phenotype, with thinner body shape compared to the control. Results are the mean ± SEM of three biological replicates with their respective three technical replications. **(B)** Triglyceride content (μmol triglyceride/μg total protein) in ds*NlVg*-treated BPHs. BPH instars (72 h after injection, *n* = 10) were used. ds*GFP* was used as the negative control. The values shown are the means ± SEM. The triglyceride content was calculated based on six biological replicates with their respective three technical replications. ^***^P < 0.001 (two-tailed unpaired *t*-test) as compared to ds*GFP*.

**Figure 8:**
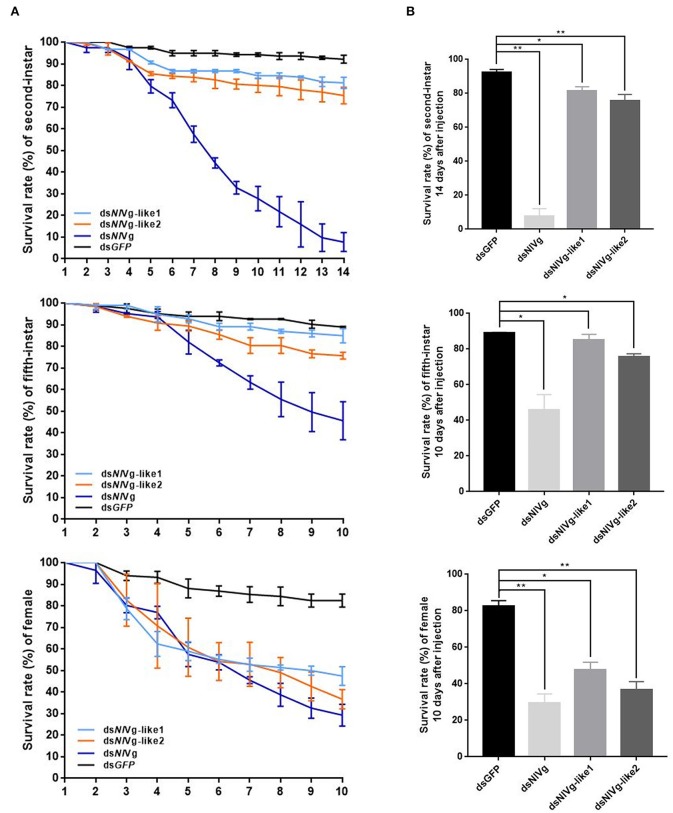
**(A)** Survival rate of BPHs 10 days after knockdown of three genes in late-instar nymphs and females (14 days after injection in early-instar nymphs). ds*NlVg-*like1, ds*NlVg-*like2, ds*NlVg*, or ds*GFP* were injected into early-stage second- and fifth-instar BPH nymphs and early-stage newly emerged female adults (0–12 h). ds*GFP* was used as the negative control for the non-specific effects of dsRNA; *n* = 100. The x-axis represents the days after injection; the y-axis represents the survival rate of the target gene. The values were calculated from three biological replicates with their respective three technical replications. (mean ± SEM). Light blue line, ds*NlVg-*like1; orange line, ds*NlVg-*like2; dark blue line, ds*NlVg*; black line, ds*GFP*. Results are the mean ± SEM of three independent experiments. **(B)** Survival rate 10 days after dsRNA injection in late-instar nymphs and females (14 days in early-instar). Results are the mean ± SEM of three biological replicates with their respective three technical replications. ^*^P < 0.05, ^**^P < 0.01 (Tukey's multiple comparisons test).

Because reduced expression levels of *NlVg*, ds*NlVg*-like1, and ds*NlVg*-like2 were also detected in the nymph stages, 0–12-h second-instar nymphs (representing early-instars) and 0–12-h fifth-instar nymphs (representing late-instars) were treated with ds*NlVg*, ds*NlVg*-like1, ds*NlVg*-like2, and ds*GFP* to determine the possible functions of *NlVg, NlVg-*like1, and *NlVg*-like2 in BPH nymphs. RT-qPCR confirmed that the dsRNAs suppressed the target genes efficiently (Figure S5A). Western blotting showed that NlVg could be weakly detected in the second-instar nymphs and was completely absent from the fifth-instar nymphs and female adults following ds*NlVg* treatment, whereas signals could be detected in all three ds*GFP*-treated groups (Figure S5B). ds*NlVg* treatment in the nymphs caused a lethal phenotype, with thinner body shapes compared with those of the control group (Figure 7A). Moreover, the ds*NlVg*-treated BPHs had significantly decreased triglyceride contents compared with the control BPHs (Figure 7B), suggesting that *NlVg* plays a vital role during the normal growth and development of the BPH. The survival rate of treated early-instar nymphs was <30% at 10 days after injection, compared with ~95% survival rate of ds*GFP*-treated BPHs (Figure 8). Intriguingly, ds*NlVg*-like1 and ds*NlVg*-like2 did not lead to high mortality rates or distinct physiological malfunctions in the nymph stages (Figure 8).

## Discussion

For several decades, Vg has been extensively studied due to its variable copy numbers and multiple functions in several oviparous species, including insects. To date, more than one *Vg* and *Vg*-like transcript has been identified in hemipteran insects, such as *B. cockerelli, Bemisia tabaci, Plautia stali*, and *Riptortus clavatus* (Shinoda et al., 1996; Ibanez et al., 2018), whereas only one *Vg* gene has been reported in *Cimex lectularius*, BPH, and cicada (Tufail et al., 2010; Nagaba et al., 2011). The present study reveals that the BPH harbors more novel *Vg* genes than previously described, which we have named *NlVg*-like1 and *NlVg*-like2. Some insects have multiple *Vg* genes with highly similar sequences and conserved domains, and they all participate in vitellogenesis. However, we found that the exon–intron organizations, conserved domains, and expression profiles of these three *Vg* genes varied greatly, which hinted that *NlVg* orthologs may have evolved different functions after duplicating. For example, the DUF1943 domain, which has been identified in Vgs from many insect species, including NlVg and NlVg-like1, was not found in NlVg-like2. DUF1943 not only interacts with lipoteichoic acid (LTA) and lipopolysaccharide (LPS) found in the outer membranes of bacteria (Sun et al., 2013), but it can also enhance macrophage phagocytosis of bacteria, playing a role in the antibacterial defense of zebrafish (Du et al., 2017). Similar cases have been described for insect Vgs, such as in *Apis mellifera* (decreased oxidative stress), *Anopheles gambiae* (anti-*Plasmodium* response), and *B. cockerelli* (immunity and lipid transportation) (Seehuus et al., 2006; Rono et al., 2010; Ibanez et al., 2018). Moreover, in animals, such as amphioxus, Vgs are considered to be immunocompetent molecules that are associated with host defense (Zhang et al., 2005; Shi et al., 2006; Li et al., 2009; Liu et al., 2009; Garcia et al., 2010); therefore, we wondered whether the different domains in NlVg play different roles in their parental molecule, allowing the protein to play dual roles in immune defense, and nutritional supply.

Vg uptake is essential for ovarian development. Previous work examining Vg or VG-like genes in hemipterous insects has primarily focused on sequence and protein structural analyses of Vg, the Vg expression pattern, phylogenetic analyses of Vg, the regulation of Vg by juvenile hormone (JH) III, 20E, or ecdysteroids, and the Vg receptor (Khalil et al., 2011; Lu et al., 2015; Ibanez et al., 2017). Functional analyses using RNAi have seldom been utilized in hemipterans. Here, we observed three different phenotypes after conducting RNAi experiments in newly emerged female BPHs. In particular, *NlVg* downregulation suppressed ovary development and stretched the fat body-filled abdomen. The phenotypes after *NlVg* RNAi may result not only from reduced Vg synthesis but also from nutritional stagnation in the fat body. A similar phenotype was found in the bedbug, *C. lectularius*, a hemipterous insect (Moriyama et al., 2016), in which hypertrophied fat bodies, atrophied ovaries and drastically reduced egg production were observed after the downregulation of *ClVg* in adult females.

Although the ds*NlVg*-like1 group had similar reproductive abilities as the control group, egg hatchability (only 65.13%) was considerably lower in the ds*GFP* group (91.41%), with 17.84% of embryos failing to break away from the eggshell in the ds*NlVg*-like1 group, compared with 1.54% in the control group, suggesting that *NlV*g-like1 may be related to the later period of embryonic development. *NlVg*-like2 knockdown decreased the number of offspring and led to lower egg hatchability compared those for other groups. The phenotypes of the abnormal eggs showed that the downregulation of these genes may result in the insufficient accumulation of Vg during oogenesis (Zhang et al., 2005; Shi et al., 2006) or may prevent lipid uptake by the ovary, affecting the nutrition and energy accumulated in the fat body, which modulates the rate of egg development (Arrese and Soulages, 2010).

Many studies have illustrated that RNAi-mediated crop protection can be considered to be a potential strategy for insect pest control. Nowadays, with the development of genome-scale high-throughput screening and the identification of homologous genes, the use of sprayed dsRNA reagents and the integration of dsRNAs that target insect genes into transgenic plants have been verified to induce RNAi effects in targeted insects. Since 1990, lepidopteran pests have been controlled by *Bacillus thuringiensis (Bt)* toxin transgenic plants (Shelton et al., 2002; Naranjo, 2011). However, no *Bt* toxin currently exists with adequate insecticidal effects against phloem sap-sucking insects, such as planthoppers, aphids and plant bugs. Hence, novel approaches to combat these sucking pests must be considered. The most destructive insect pest for rice crops, BPHs damage rice directly by sucking plant sap, which can transmit viruses, and they have the robust capacity to produce offspring. When BPH nymphs were fed with rice plants that express dsRNAs, RNAi of targeted genes could be triggered and the gene transcription levels were suppressed, although no remarkable lethal phenotypes were observed (Zha et al., 2011), suggesting that suitable target genes should be strictly selected when designing dsRNA-transgenic plants. *NlVg* and *NlVg*-like genes may impact the strategies used by the agricultural industry to combat crop pests, such as BPH, because these genes are critical for reproduction, oocyte maturation and nymph development. The identification of these genes could be of tremendous value for future RNAi applications via the development of transgenic plants that target phloem feeders. Moreover, these genes may provide potential targets for BPH management. RNAi-mediated crop protection could not only represent a potential novel technology for pest control but could also be used as a complementary approach to other control methods. However, further intensive studies are required to improve the efficiencies of dsRNA applications mediated by oral delivery and dsRNA spraying methods to increase the feasibility of RNAi-mediated crop protection techniques.

In conclusion, NlVg is a conventional insect Vg that is involved not only in egg yolk formation and supporting embryogenesis, but also in the normal development of BPH nymphs; the *NlVg*-like1 gene plays an important role in late embryogenesis, ensuring that individuals hatch properly, and the *NlVg*-like2 gene may play roles in oocyte nutrition absorption and in embryonic development. Nevertheless, the specific mechanism behind the *NlVg*-like1 and *NlVg*-like2 genes in development requires further research. These results suggest the potential of NlVg and Nl*Vg-like* genes as the promising target for RNAi-based population management of the BPH.

## Data Availability

The datasets for this manuscript are not publicly available because the genetic sequences studied in this manuscript have been submitted to GenBank and will be released to the public database as soon as they are processed. Requests to access the datasets should be directed to C-XZ, chxzhang@zju.edu.cn.

## Author Contributions

C-XZ and YS designed the experiments. YS carried out the main experiments. Y-ZC helped to perform the experiments. All authors contributed to critical analysis. YS and Y-HL analyzed experimental results. YS and C-XZ wrote the manuscript and all authors approved the final manuscript for publication.

### Conflict of Interest Statement

The authors declare that the research was conducted in the absence of any commercial or financial relationships that could be construed as a potential conflict of interest.

## Supplementary Material

Figure S1 Nucleotide and deduced amino acid sequence of the *Nl*Vg coding region. Blue, the location of primers designed for RT-qPCR; red, the location of primers designed for dsRNA synthesis; yellow frame, the position of lipoprotein N-terminal domain (LPD_N) domain; blue frame, unknown function motif 1943 (DUF1943) domain; greed frame, Von Willebrand factor type D domain (VWD); red frame, the site of the promoter or terminator.

Figure S2 Nucleotide and deduced amino acid sequence of the *Nl*Vg-like1 coding region. Blue, the location of primers designed for RT-qPCR; red, the location of primers designed for dsRNA synthesis; yellow frame, the position of lipoprotein N-terminal domain (LPD_N) domain; blue frame, unknown function motif 1943 (DUF1943) domain; greed frame, Von Willebrand factor type D domain (VWD); red frame, the site of the promoter or terminator.

Figure S3 Nucleotide and deduced amino acid sequence of the *Nl*Vg coding region. Blue, the location of primers designed for RT-qPCR; red, the location of primers designed for dsRNA synthesis; yellow frame, the position of lipoprotein N-terminal domain (LPD_N) domain; blue frame, unknown function motif 1943 (DUF1943) domain; greed frame, Von Willebrand factor type D domain (VWD); red frame, the site of the promoter or terminator.

Figure S4 Exon–intron organization in 3 *Nilaparvata lugens* vitellogenin (*Nl*Vg) and vitellogenin-like genes (*Nl*Vg*-*like1 and *Nl*Vg*-*like2).

Figure S5 Effection of RNA interference. **(A)** The values effect of RNA interference using the dsRNA molecules targeting the specific regions of *Nl*Vg*, Nl*Vg*-*like1, and *Nl*Vg*-*like2. BPHs were treated dsRNAs at early-instar (second-instar), late-instar (fifth-instar) nymphs and female; *n* = 100. Real-time qPCR analysis of dsRNA-treated insects showed that the target gene transcripts were inhibited effectively 3 days after injection (*n* = 10). Double-stranded RNA for green fluorescent protein were injected in the control group. *N. lugens* 1*8S* rRNA was used as the internal control genes. Mean ± SEM from three biological replicates with their respective three technical replications. ^*^*P* < 0.05, ^**^*P* < 0.01, ^***^*P* < 0.001 (two-tailed unpaired *t*-test). **(B)** Western blotting analysis of *Nl*Vg after ds*Nl*Vg or ds*GFP* RNAi treatment at the early-instar (2nd-instar), late-instar (5th-instar) nymphs and females. ~8 μg of protein from each nymph sample, and 4 μg of protein from female adult sample was used. β-actin was used as the positive control.

Table S1 Primers used in this work.

Table S2 Sequences used in the phylogenetic analysis.

Video S1 The hatching process of offspring in dsNlVg-like1-treated group.
