# Supplementary material for: Vitellogenin and Vitellogenin-Like Genes in the Brown Planthopper
Source: Front Physiol. 2019 Sep 18;10:1181. doi: 10.3389/fphys.2019.01181 (PMC6759490; doi:10.3389/fphys.2019.01181)
Supplement: Figure S3 — Nucleotide and deduced amino acid sequence of the NlVg coding region. Blue, the location of primers designed for RT-qPCR; red, the location of primers designed for dsRNA synthesis; yellow frame, the position of lipoprotein N-terminal domain (LPD_N) domain; blue frame, unknown function motif 1943 (DUF1943) domain; greed frame, Von Willebrand factor type D domain (VWD); red frame, the site of the promoter or terminator. [file Data_Sheet_3.PDF]

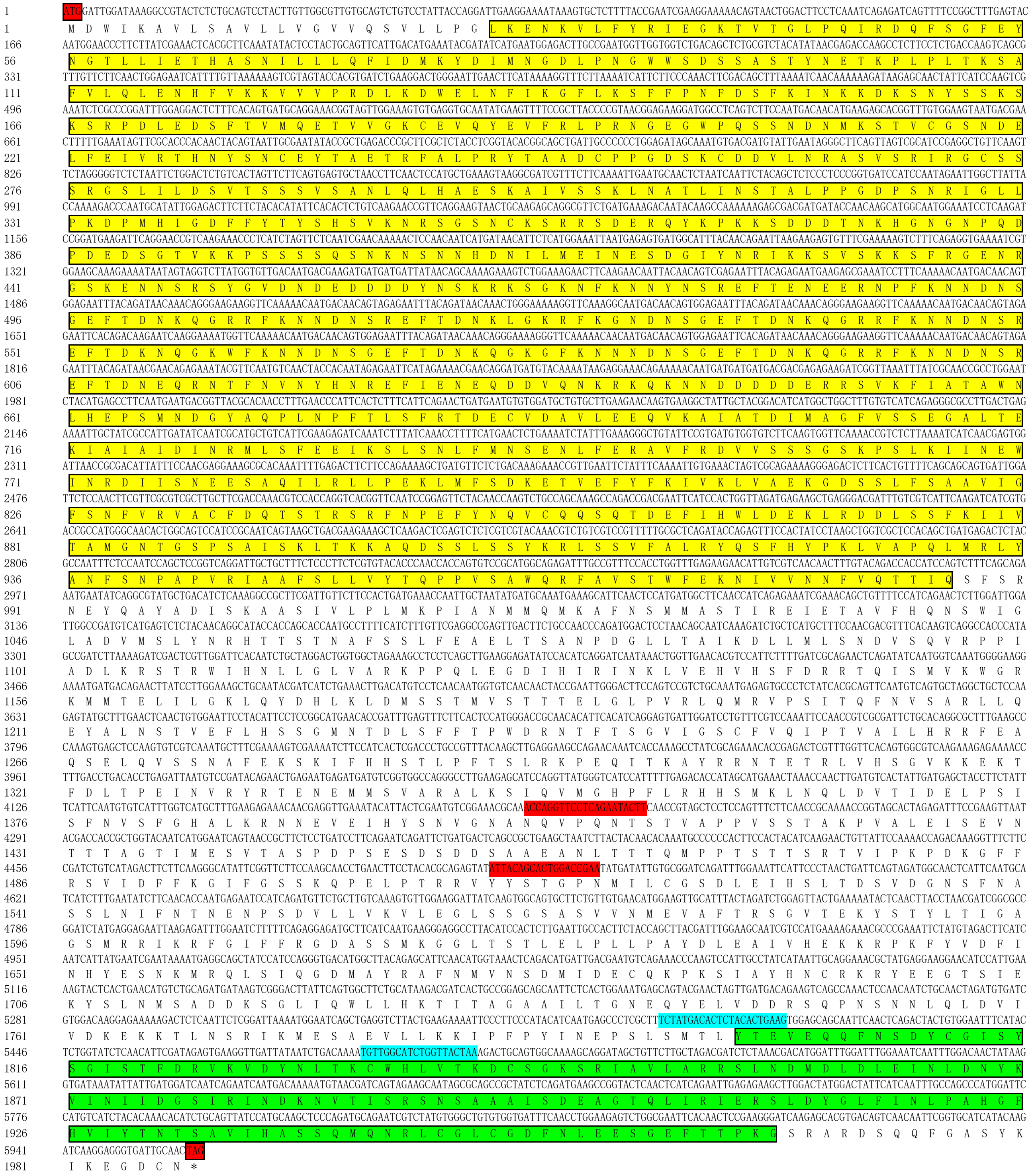

Figure S3. Nucleotide and deduced amino acid sequence of the *Nl*Vg-like2 coding region. Blue, the location of primers designed for RT-qPCR; red, the location of primers designed for dsRNA synthesis; yellow frame, the position of lipoprotein N-terminal domain (LPD_N) domain; blue frame, unknown function motif 1943 (DUF1943) domain; greed frame, Von Willebrand factor type D domain (VWD); red frame, the site of the promoter or terminator.
