# Supplementary material for: Vitellogenin and Vitellogenin-Like Genes in the Brown Planthopper
Source: Front Physiol. 2019 Sep 18;10:1181. doi: 10.3389/fphys.2019.01181 (PMC6759490; doi:10.3389/fphys.2019.01181)
Supplement: Figure S4 — Exon–intron organization in 3 Nilaparvata lugens vitellogenin (NlVg) and vitellogenin-like genes (NlVg-like1 and NlVg-like2). [file Data_Sheet_4.PDF]

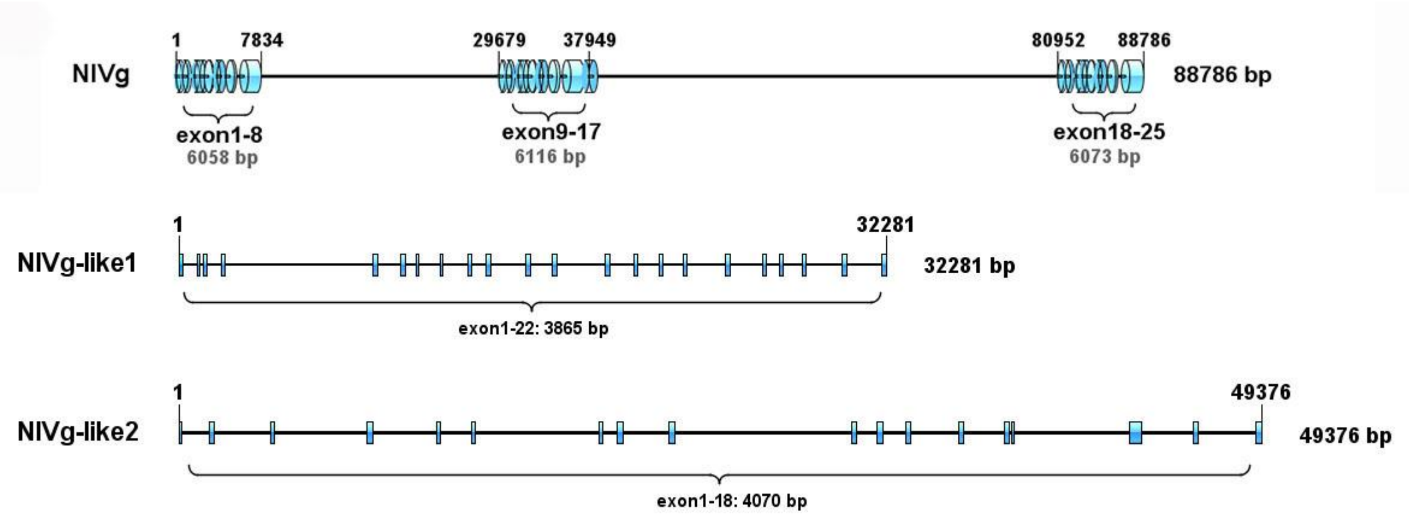

Figure S4. Exon−intron organization in 3 *Nilaparvata lugens* vitellogenin (*Nl*Vg) and vitellogenin-like genes (*Nl*Vg-like1 and *Nl*Vg-like2).
