# Supplementary material for: Vitellogenin and Vitellogenin-Like Genes in the Brown Planthopper
Source: Front Physiol. 2019 Sep 18;10:1181. doi: 10.3389/fphys.2019.01181 (PMC6759490; doi:10.3389/fphys.2019.01181)
Supplement: Figure S5 — Effection of RNA interference. (A) The values effect of RNA interference using the dsRNA molecules targeting the specific regions of NlVg, NlVg-like1, and NlVg-like2. BPHs were treated dsRNAs at early-instar (second-instar), late-instar (fifth-instar) nymphs and female; n = 100. Real-time qPCR analysis of dsRNA-treated insects showed that the target gene transcripts were inhibited effectively 3 days after injection (n = 10). Double-stranded RNA for green fluorescent protein were injected in the control group. N. lugens 18S rRNA was used as the internal control genes. Mean ± SEM from three biological replicates with their respective three technical replications. *P < 0.05, **P < 0.01, ***P < 0.001 (two-tailed unpaired t-test). (B) Western blotting analysis of NlVg after dsNlVg or dsGFP RNAi treatment at the early-instar (2nd-instar), late-instar (5th-instar) nymphs and females. ~8 μg of protein from each nymph sample, and 4 μg of protein from female adult sample was used. β-actin was used as the positive control. [file Data_Sheet_5.PDF]

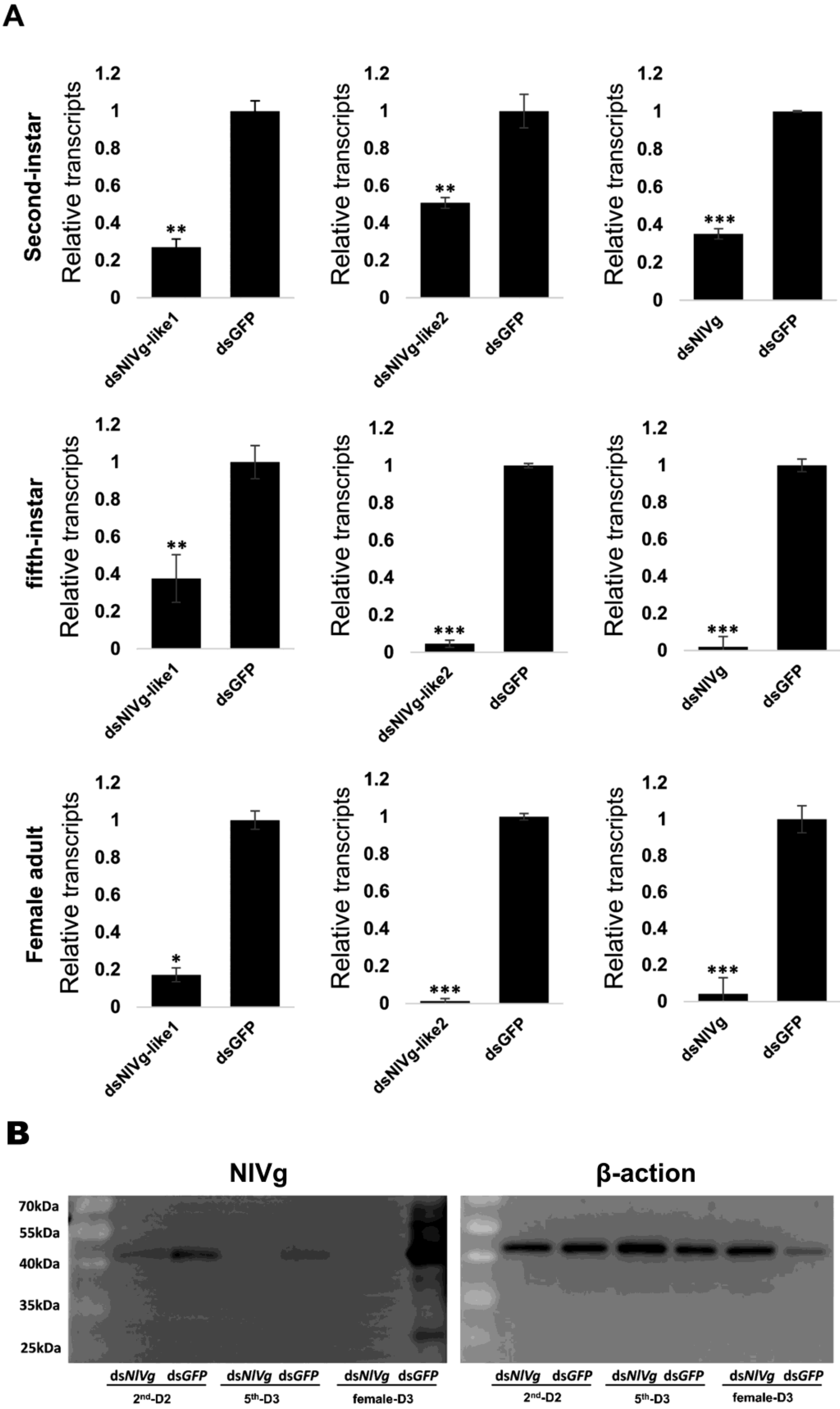

Figure S5. Effection of RNA interference. (A) The values effect of RNA interference using the dsRNA molecules targeting the specific regions of *Nl*Vg, *Nl*Vg-like1 and *Nl*Vg-like2. BPHs were treated dsRNAs at early-instar (second-instar), late-instar (fifth-instar) nymphs and female. Real-time qPCR analysis of dsRNA-treated insects showed that the target gene transcripts were inhibited effectively 3 days after injection (n=10). Double-stranded RNA for green fluorescent protein were injected in the control group. *N. lugens* 1*8S* rRNA was used as the internal control genes. Mean±SEM from three independent experiments. **P*<0.05, ***P*<0.01, ****P*<0.001 (two-tailed unpaired t-test). **(B)** Western blotting analysis of *Nl*Vg after ds*Nl*Vg or ds*GFP* RNAi treatment at the early-instar (2nd-instar), late-instar (5th-instar) nymphs and females. Approximately 8 μg of protein from each nymph sample, and 4 μg of protein from female adult sample was used. β-actin was used as the positive control.
