# Supplementary material for: Vitellogenin and Vitellogenin-Like Genes in the Brown Planthopper
Source: Front Physiol. 2019 Sep 18;10:1181. doi: 10.3389/fphys.2019.01181 (PMC6759490; doi:10.3389/fphys.2019.01181)
Supplement: Table S1 — Primers used in this work. [file Table_1.DOCX]

**Table S1.** Primers used in this work.

| **Primer usage** | | **Primer name** | **Primer sequence (5'- 3')** | **Concentration** |
| --- | --- | --- | --- | --- |
| **cloning** | *NlVg* | NlVg-1-F | CGGACTAACTCTGATTCTCTGC | 10mM |
|  |  | NlVg-1-R | GTGTGCCAACTACTCCCTTCT | 10mM |
|  |  | NlVg-2-F | ACCCTTGTCTCCGTGTCAC | 10mM |
|  |  | NlVg-2-R | CTTGAATGCTTCCCAACTCT | 10mM |
|  |  | NlVg-3-F | CACTCACCTCCTTCACCATT | 10mM |
|  |  | NlVg-3-R | GAGCTTAATTGGCTTTCGTAG | 10mM |
|  |  | NlVg-4-F | GAAGCTCCCTGTCCAAGA | 10mM |
|  |  | NlVg-4-R | TCATCCGAGTTGTCAGCA | 10mM |
|  |  | NlVg-5-F | TGAACTTTGGCAAGGAG | 10mM |
|  |  | NlVg-5-R | ATTGTGGTATGGGTAGGC | 10mM |
|  |  | NlVg-6-F | GCCTACCCATACCACAAT | 10mM |
|  |  | NlVg-6-R | CAAGCAAGACATATCCACC | 10mM |
|  | *NlVg-like1* | NlVg-like1-1-F | ACTTTATTGTGCTTTCGTGAG | 10mM |
|  |  | NlVg-like1-1-R | GATTGGTAGGGCTGTAGTG | 10mM |
|  |  | NlVg-like1-2-F | TCACAGTGAAAGGACGTATATT | 10mM |
|  |  | NlVg-like1-2-R | ATGATGACATTGGGTAGGC | 10mM |
|  |  | NlVg-like1-3-F | TACCATACAGTCACTCACCCAG | 10mM |
|  |  | NlVg-like1-3-R | AGTCAAGAGCGGCACAAA | 10mM |
|  |  | NlVg-like1-4-F | AGTTGCCCTTCTTGGTCTG | 10mM |
|  |  | NlVg-like1-4-R | GTTAATCTACTTCGTTGCGTTT | 10mM |
|  | *NlVg-like2* | NlVg-like2-1-F | ttatctgcttgatgctttc | 10mM |
|  |  | NlVg-like2-1-R | TTTTTGGCTTGTATTGTCTT | 10mM |
|  |  | NlVg-like2-2-F | CCGAGGCTGTTCAAGTT | 10mM |
|  |  | NlVg-like2-2-R | TGGACTGCCAGTGTTGC | 10mM |
|  |  | NlVg-like2-3-F | CATCCGCAATCAGTAAGC | 10mM |
|  |  | NlVg-like2-3-R | AAGTCCCAATTCGGTAGTT | 10mM |
|  |  | NlVg-like2-4-F | TGCTAGGACTGGTGGCT | 10mM |
|  |  | NlVg-like2-4-R | GAACCTGGTTTGCGTTT | 10mM |
|  |  | NlVg-like2-5-F | AGTGGCGTCAAGAAAGAG | 10mM |
|  |  | NlVg-like2-5-R | GGCAATGGACTTGGGTT | 10mM |
|  |  | NlVg-like2-6-F | AAAGAAACGCCCGAAAT | 10mM |
|  |  | NlVg-like2-6-R | GCAATCACCCTCCTTGA | 10mM |
| **double stranded RNA synthesis** | | T7NlVg-F | TAATACGACTCACTATAGGGAGAGACTGTTTGCAGCCACCTA | 10mM |
|  |  | T7NlVg-R | TAATACGACTCACTATAGGGAGACCGTTGCGGATTTGATT | 10mM |
|  |  | T7NlVg-like1-F | TAATACGACTCACTATAGGGAGATCAGCCAGCAGTCCCTCT | 10mM |
|  |  | T7NlVg-like1-R | TAATACGACTCACTATAGGGAGAGTTTTCCACTCAATGTCGGTA | 10mM |
|  |  | T7NlVg-like2-F | TAATACGACTCACTATAGGGAGAACCAGGTTCCTCAGAATACTT | 10mM |
|  |  | T7NlVg-like2-R | TAATACGACTCACTATAGGGAGATTTGGTCCAGTGCTGTAAT | 10mM |
|  |  | T7GFP-F | TAATACGACTCACTATAGGGAGAATGAGTAAAGGAGAAGAACTTTTC | 10mM |
|  |  | T7GFP-R | TAATACGACTCACTATAGGGAGATTTGTATAGTTCATCCATGCCATGT | 10mM |
| **quantitative real-time PCR** | | qVg-F | CACTGCCCGTGCTGTGCTCTA | 10mM |
|  |  | qVg-R | TGACTTCCTTGCTTTGCTCCC | 10mM |
|  |  | qNlVg-like1-F | GCTGACTCTGCTGATTCT | 10mM |
|  |  | qNlVg-like1-R | GCGTGTAACAAGGATAGGT | 10mM |
|  |  | qNlVg-like2-F | TCTATGACACTCTACACTGAAG | 10mM |
|  |  | qNlVg-like2-R | TTAGTAACCAGATGCCAACA | 10mM |
|  |  | 18S-F | GTAACCCGCTGAACCTCCT | 10mM |
|  |  | 18S-R | TCCGAAGACCTCACTAAATC | 10mM |
