# Supplementary material for: Vitellogenin and Vitellogenin-Like Genes in the Brown Planthopper
Source: Front Physiol. 2019 Sep 18;10:1181. doi: 10.3389/fphys.2019.01181 (PMC6759490; doi:10.3389/fphys.2019.01181)
Supplement: Figure S1 — Nucleotide and deduced amino acid sequence of the NlVg coding region. Blue, the location of primers designed for RT-qPCR; red, the location of primers designed for dsRNA synthesis; yellow frame, the position of lipoprotein N-terminal domain (LPD_N) domain; blue frame, unknown function motif 1943 (DUF1943) domain; greed frame, Von Willebrand factor type D domain (VWD); red frame, the site of the promoter or terminator. [file Data_Sheet_1.PDF]

```
1     ATGAACGGACTAACTCTGATTCTCTGCGTCATCGCGGTTGCCGGAGTATCTGCCAGCGGCAGTGGACCATGGAACAGCAACCAGCAGTACCGCTACCATGTGCAAGGCAGATCACTGTCAGCCATGCACCAGTCAGGATCAAACCAGTACGTGGGCATGCACCTG
1     M N G L T L I L C V I A V A G V S A S G S G P W N S N Q Q Y R Y H V Q G R S L S A M H Q S G S N Q Y V G M H L
166   AGAGCAGAATTGGAAGTTGAGGCGAAGAATGAGAACCAGGCTGTGTTCAAGATCTCCAAGGCCGAGTACGCCGATGTGCACCAGAACCTAAGTGGAGGCTGGCAACAAGAGTTACGCAGCAATGAGCTGCAGTACAAGCAGTTGCCCCTGTCGCAAGCAAACCAA
56    R A E L E V E A K N E N Q A V F K I S K A E Y A D V H Q N L S G G W Q Q E L R S N E L Q Y K Q L P L S Q A N Q
331   GTCTTTCAAGTCAACTACAAGCAGGGAGCAGTGCGCAGTCTGCAGGTCAACAGGAACACACCCACCTGGGAACTGAACATGATCAAGGGATTCGTCAGCCTGTTCCAGGTCGATGTGACTGGCCAGAATGCCATCAAGTCTAGGCGCAACATTGTGCCCAATGGC
111   V F Q V N Y K Q G A V R S L Q V N R N T P T W E L N M I K G F V S L F Q V D V T G Q N A I K S R R N I V P N G
496   CAGCAGGTGAGTGGCTCGTTCAAGGTTATGGAGGACTCTGTCACCGGCAAGTGTGAGACCCACTACGATGTTGATGAGCTGCCAATGAGAGTTGTGCAACAGCACCCAGAGATTGCACCACTTGCAGTTAAGCAGCAGGGCCAGGGCCAGGGCCAGAGCCACAGC
166   Q Q V S G S F K V M E D S V T G K C E T H Y D V D E L P M R V V Q Q H P E I A P L A V K Q Q G Q G Q G Q S H S
661   CGTCTCATTCAGGTGGTCAAGTCAAGGAACTTTAGCAACTGTGATAACCCAGTCACCTACCACTTTGGATTCACTCAGGAAAGCAACTTTGAGCCAGCCAGCAACCAGATGGGCAACCTTGTCAGCCGTGCCGCAATGGGCCGTATCATTATTGCTGGAGAACCA
221   R L I Q V V K S R N F S N C D N P V T Y H F G F T Q E S N F E P A S N Q M G N L V S R A A M G R I I I A G E P
826   GACAGTTACACCATCCACTCATCGGTCACTCAGAATGAGATTGCCATCAGTCCATTCGGCTACAACCAGCAGAAGGGAGTAGTTGGCACACTCATGAATGCCACCCTTGTCTCCGTGTCACATGCCTCTTCTGGCTCTCCTCAGTCAGTCCAGAACCCACAGAAA
276   D S Y T I H S S V T Q N E I A I S P F G Y N Q Q K G V V G T L M N A T L V S V S H A S S G S P Q S V Q N P Q K
991   ATCAACGATCTGGTTTATGAGTTCAACCCAGCATCAAACAGTGAGAGCAACCAAAGATCTAGCCACTACACCAGGCAGCAGGCTGATAATGAAGATGACAGCAGCTCAAGCTCATCTAGCAGTGACTCTTCTTCATCATCCTCCTCCTCTTCATCTAGCTCATCA
331   I N D L V Y E F N P A S N S E S N Q R S S H Y T R Q Q A D N E D D S S S S S S S S D S S S S S S S S S S S S S
1156  TCATCATCGAGCAGCTCAGAGGAGAACAACAAGAATAGTAAGAAGAACAACAACAACAAGAACTGGAACAACAAGAACCAGAAGAAGAAGAACAACAACAACAGGAACAACCATAATGACAATGACAACAACCAGGATAACTCCAATGAAAACAATAATGATGAT
386   S S S S S S E E N N K N S K K N N N N K N W N N K N Q K K K N N N N R N N H N D N D N N Q D N S N E N N N D D
1321  GCTTACTGGAGGAGCCAGCAGAAGACCAAGTTCAGGTCACGCAGGAGTATTCTGAGGAACTACAACAACGAGAATGATGATGACAACAACCAAAACAGGAACCAGAACAGAAACAACAACAACAACAACAACAATGACTCCTCTGAAGAGTGCAATGAAAACCAG
441   A Y W R S Q Q K T K F R S R R S I L R N Y N N E N D D D N N Q N R N Q N R N N N N N N N N D S S E E C N E N Q
1486  AACACCAATAACAAGCACAACAACAACAAGAACTGGAACAACAATAACAATAACAATAACAAGAACTGGAACAACAATGACAACAAGAACTGGAACAACAATGACAACAACAACAAGAACAGGAACAACAATGACAATGATGACTCATCATCAAGCTCAAGCAGT
496   N T N N K H N N N K N W N N N N N N N N K N W N N N D N K N W N N N D N N N K N R N N N D N D D S S S S S S S
1651  TCCAGTTCAAGCTCCTCCTCCTCGTCCTCATCTTCATCCTCTTCGTCCTCATCCTCTGATCTTGACAGCAGCGAAGAGAACTGGCAGCAGAAGCCTGGCATGAATGATGCACCAAGAACACCTTTCTTGCCCCACTTTGTTGGAGTAAGAGGCAACTCAATTCAG
551   S S S S S S S S S S S S S S S S S S S D L D S S E E N W Q Q K P G M N D A P R T P F L P H F V G V R G N S I Q
1816  GCTGACAAGCAGGTCGACATTGTCAATGAAGTCCAAAAGGTAGCCATGAGGATTGGCGCCCAGGTCCAGAGACCCAGTGCCATTCCAGGACAGAACACACTCACCTCCTTCACCATTCTCACCAGGATGATTCAGACCATGTCAGCCAAGCAGATCCAGGAAGTC
606   A D K Q V D I V N E V Q K V A M R I G A Q V Q R P S A I P G Q N T L T S F T I L T R M I Q T M S A K Q I Q E V
1981  AAGCAGAGACTTTTCATTGACAGGAACAATGCCAATGGCAAAAGTTCTGCTGATGCCAAGAAACTCCAGAGTTGGGAAGCATTCAAGCATGCTACAGCTAATGCTGGAACTGGACCAGCATTGGAAGCCATCAAGAACTGGGTTGAAAAGGGTGATGTGAGGAAT
661   K Q R L F I D R N N A N G K S S A D A K K L Q S W E A F K H A T A N A G T G P A L E A I K N W V E K G D V R N
2146  GAGAAGGCAGCTGAACTTGTTGCTGTTCTGCCAAGAACCGCCAGACTGCCAACTGACCAGTACATCAAGACTTTCTTCCAATTTGCCACCTCATCAAATGTGCAGAACCAGAAGTACCTCAACTCCACCATCATCCTTGGATTCTCTGAAATCCTGAGGAAAGCT
716   E K A A E L V A V L P R T A R L P T D Q Y I K T F F Q F A T S S N V Q N Q K Y L N S T I I L G F S E I L R K A
2311  CAAGTTGACTCAGACACCAAACACATGCGCTTTGGAGTTCACAGCTTTGGTCACTTGACATCCAAGCATGATCAATCCCTGCATCAGGAATACATGCCCTACTTGGAAGAGAAACTCAAGAGTGCCTTTGAAAAGGGTGATAGCCAGAAAATCATTGTCTACATC
771   Q V D S D T K H M R F G V H S F G H L T S K H D Q S L H Q E Y M P Y L E E K L K S A F E K G D S Q K I I V Y I
2476  CAAGCTCTTGGAAACACTGCACACCCAAGACTATTGAAGACCTTTGAACCCTACCTGGAAGGAAAGAAATCAGCATCACGCTTCCAGCGCCTCTTGATGGTTGCCAGCCTCTACCAGATGACCCGTGTCCACCCAACCACTGCCCGTGCTGTGCTCTACAGAATC
826   Q A L G N T A H P R L L K T F E P Y L E G K K S A S R F Q R L L M V A S L Y Q M T R V H P T T A R A V L Y R I
2641  TACAAGAACCCAGGAGAAGCCGCCGAACTCAGAGTGGCTGCCCTACACCTTTTGGCCAATGCTAACCCATCTGCAGCCATGCTACAGAGGATGGCCCAGCAGACCCACTGGGAGCAAAGCAAGGAAGTCATCTCAGCTACTCAATCCTTCATCAAGAGTGCTGCT
881   Y K N P G E A A E L R V A A L H L L A N A N P S A A M L Q R M A Q Q T H W E Q S K E V I S A T Q S F I K S A A
2806  CGTATGGATCAGAATCCCAACTCCATTGAATTGGCAAGGAATGCTCAGGCAGCTGTTGACATGCTCAACCCCAATGAGTATGGAAGCTCCCTGTCCAAGAACTTCCTCAGCTCATTTGTCATTGACAACATTGACAAGAGCTACGAAAGCCAATTAAGCTCCATT
936   R M D Q N P N S I E L A R N A Q A A V D M L N P N E Y G S S L S K N F L S S F V I D N I D K S Y E S Q L S S I
2971  GGCAGTGTTGACAGTATTATTCCAAGCAGTGTGTTTGTCAACTTCATGGCCAATGATGGAGGATACAAGCACCAAGTGTTCCATCACTCTGCTATGTTCTCCAGTGTTAATGACCTTTTGGAGCTTGTGAACACTCAATTCAAGAACAACAACAACAACAACCGC
991   G S V D S I I P S S V F V N F M A N D G G Y K H Q V F H H S A M F S S V N D L L E L V N T Q F K N N N N N N R
3136  AGAAACAACAAGTCTGGATCTCATGATAATGAAGACAACCATAACAACAGGAACTCAAACAACGAATGGACAGCTGAGAATGTTCTCAAGGCCCTCAACATCCAGAAGGACCAGGCTGAACAGCTGGAAGGAAACTTCTTCCTCACCATGCTGGGAGGAAAGCGT
1046  R N N K S G S H D N E D N H N N R N S N N E W T A E N V L K A L N I Q K D Q A E Q L E G N F F L T M L G G K R
3301  GCTTTTGCCATCAACAACCACACCATGAGAAGATCCCCATCCATTTTCAAGGAAGCAGCACAGAAACTGAAGCACACCTCATTCAACCTGACTCAGTTCTACAGCAAGAACACAATGAAAGTAGCCTTCCCCACCCCAATGGGTCTGCCTTTTGTCTATACCTCC
1101  A F A I N N H T M R R S P S I F K E A A Q K L K H T S F N L T Q F Y S K N T M K V A F P T P M G L P F V Y T S
3466  AGTGTACCCACCATGGTGTATGTAGGTGGAGAGACCAAGGTCAACTCTCACCCAGACTTGGCCAATGGCAACAACAACTTCGTCAACATTCCTCAATACATCAACATCTCAGCTGACATTGAAGCAGTCTACTCAATGCAGTCCAACAGCAAATTTGGTATGGTT
1156  S V P T M V Y V G G E T K V N S H P D L A N G N N N F V N I P Q Y I N I S A D I E A V Y S M Q S N S K F G M V
3631  GCTCCATTCAACCACCATGAGTACTATGCCAGTGTTGAGAAGAACATGCAATTCTATATGGCTGTTCAGACAGAGGCAAACATTGACTTGGAAAACAACGAGGTCGAATTCACAGTGCAGCCACTCAACAAGGAAGACAAGCAGAATGTGTTCCAGTACAGCTCA
1211  A P F N H H E Y Y A S V E K N M Q F Y M A V Q T E A N I D L E N N E V E F T V Q P L N K E D K Q N V F Q Y S S
3796  GTGCTCTACACCACCAAGTCAAACATCCTCAACTTCAACCCAAATAACCAGGAGGATGGCACTGAGAGAGTGCATGTTGGCAAAGCTAAACAGATCCAGATGAACTTTGGCAAGGAGAGCACTGGATTCGCATTTGAGGCCAGCTACTGGAGTGAGAATGGCTTT
1266  V L Y T T K S N I L N F N P N N Q E D G T E R V H V G K A K Q I Q M N F G K E S T G F A F E A S Y W S E N G F
3961  GGAGACTTTGCCACCCTCTACAATGAAGTTAGCAAGTTTGATTTCCAGTCTGCAATGACATCTCCCTGGGCTCAAGGCTCCATCAACTCCAACAACATTACTGTTGCCTTCAATCCAAGGCAGAGCACCTCTCAGGTTGCCAAGTTTACTTTCTCCTATGCTGAC
1321  G D F A T L Y N E V S K F D F Q S A M T S P W A Q G S I N S N N I T V A F N P R Q S T S Q V A K F T F S Y A D
4126  AACTCGGATGACAACAATAACTCCCACAGTGGACATGACAGCAACAACAGCAACAACAACAATAACAACAGAGCAGACTACTCTGATGCTCAGCCATCTTCCACAACAGGGCAGACTACTCTGATGCTCAGCCATCTTCCACTGCTGCCAACAGTCGTTTCCAGA
1376  N S D D N N N S H S G H D S N N S N N N N N N R A D Y S D A Q P S S T T G Q T T L M L S H L P L L P T V V S R
4291  CAGAATGAATTCCTGCGCAAGGCTGCTGCTGGCATCTCAGGTGCTGATGCTATGGTTGTTGATGTGTCTGCTAGATTCCAAGACAGCCATGGTCAATCCAATGCCCAGTATGTAGCCACTGTAGCAATGGCCAACAGTGATGCTTCTCCAAACGCTCGTATGCTG
1431  Q N E F L R K A A A G I S G A D A M V V D V S A R F Q D S H G Q S N A Q Y V A T V A M A N S D A S P N A R M L
4456  TTCTTTGCATCAATGAACCCAGCTAACTCTGACTCCAAGGCCCAGGTGTGTGCTGCAGCTGCCAGTAACTTCCCCAATGTGCCACTCATGAACTTCCATGATGCCCTCAAGGCAAACCCAACTTCCCGCATCAGTGCTGACATTGCCTTTGGAGCACAATGCAAT
1486  F F A S M N P A N S D S K A Q V C A A A A S N F P N V P L M N F H D A L K A N P T S R I S A D I A F G A Q C N
4621  GCTGGAGGACACATCCATGCTGATGCCAAGTTGTCACAGACCCAGGAATTCCAGGAGTATGCAAAGAGCCGTCCAATGGCCAAGAAATGCTTCCAGTTGATGGAGAAGGGCCAGGCTCTTGAATATGCTTGCCAGAATGCCACCAAGGTCGCCAACATGCTCAAC
1541  A G G H I H A D A K L S Q T Q E F Q E Y A K S R P M A K K C F Q L M E K G Q A L E Y A C Q N A T K V A N M L N
4786  AACTACGAAGTCTCTGTCAAGTATGATAGAGTATCAAGTGTCTTCAAGAACGTCACCTACAGCATCTACTCAGCTCTGGCTCAAGCTGCCTACCCATACCACAATGAAAACATGTTCAGCCAGAACAGCAATCCTTCTGGCAAGATTGATCTCAATGCCAGGTTC
1596  N Y E V S V K Y D R V S S V F K N V T Y S I Y S A L A Q A A Y P Y H N E N M F S Q N S N P S G K I D L N A R F
4951  AACTACAACCTCCGTTACTTCAATGCTTCAATCAACACTCCATTCTTCTCTGCCAATGTCAAGAATGTTGAAGTACACCATGCTCTCAGACCTCTGGTGATCTTCCATCCATCACTCAACTCACTTGAGCACTTGAGCTCATGTCCTACAATGAGAACTACGATT
1651  N Y N L R Y F N A S I N T P F F S A N V K N V E V H H A L R P L V I F H P S L N S L E H L S S C P T M R T T I
5116  ACCCAACATGTTCTGTCAGCAAGAACTCCATCAGCACATTCGACAACAAGACCTACTCATGCTGACCTTGAAGGCTGGCATGTGATGTTTGCCTCCACTCCAAAGAACTTCAACGACAACTCTGGAAGATACAGTGCCAGCAACAGCCAGTCCAACAGCTTCTAC
1706  T Q H V L S A R T P S A H S T T R P T H A D L E G W H V M F A S T P K N F N D N S G R Y S A S N S Q S N S F Y
5281  AAGTACAAGAAGGTTGTTGTCTTGGCCAAGAACGCCGGATCTCAGCGCAAGGCTGTCAAGATGCTGTTGGGAGAGAATGTCATTGACATCAACCCCTCTGGCTCCGAGTCAAGTGACAACAGCCCCAACGCCAATGTCCAGGTCAATGGAAACAAGGTGCAGATT
1761  K Y K K V V V L A K N A G S Q R K A V K M L L G E N V I D I N P S G S E S S D N S P N A N V Q V N G N K V Q I
5446  GCCAACAACAGGATGGCCAGTTTCGATGACTTTGATGGTGAGACCCTCGTTGAGATCTCTGTCACAGACAATGGAGAGGTGCAGGTTCAGTCTTCATCCCATGGCATTGCTGTCTACCACGATGGTGCCAACTTCATCATTGATGCTGACAGCTACCACAGAGGT
1816  A N N R M A S F D D F D G E T L V E I S V T D N G E V Q V Q S S S H G I A V Y H D G A N F I I D A D S Y H R G
5611  GAGGTGCGTGGTCTCTGCGGTACCTACTCTGGTGACAAGTACTCCCCCCTTCACCCGGAAAAAAACAAGTGCATTATGAGGGAGGCTATTCCGTTTGCAGCCACCTATGCTCTGCCAGGCAGCAGCAACAGCAACGTTGAACAGCTCAAGAGGCAGGCTGACCAG
1871  E V R G L C G T Y S G D K Y S P L H P E K N K C I M R E A I P F A A T Y A L P G S S N S N V E Q L K R Q A D Q
5776  ATGACTTGCTTCAGGCGCCGTCACATCTTTGCTAATGTCATTACTTCCAACGACTATGACAGAAGCAGCAGCAGCAGCAGCAGCAACAGGAACAACAACAGGAACAACAACAAGAACAACAGGAGCAACAACAACAGTTCAGAGAGACTCGCAAACCCAACCAAG
1926  M T C F R R R H I F A N V I T S N D Y D R S S S S S S S N R N N N R N N N K N N R S N N N S S E R L A N P T K
5941  CTTATCCAGGACGTCAAGAACAATGGTGACCAAGTCTGCATCAGTATTCGCCCAGTCCCCAAGTGCCAGAAAGGCTTCTCCCCAGCTGGATCATCTGAGAAAGAGGTTGACTACGTCTGTATGAGTCACGGCAAGAATGCCCAATTTTGGATCAATCAGATCTTC
1981  L I Q D V K N N G D Q V C I S I R P V P K C Q K G F S P A G S S E K E V D Y V C M S H G K N A Q F W I N Q I F
6106  CAAGGTGGATATGTCTTGCTTGAACAGAAGCAGCATAATGCCACATTCATGAAGAACATTCCACAGAGATGTGTCAGAGACAACTAA
2036  Q G G Y V L L E Q K Q H N A T F M K N I P Q R C V R D N *
```

Figure S1. Nucleotide and deduced amino acid sequence of the *Nl*Vg coding region. Blue, the location of primers designed for RT-qPCR; red, the location of primers designed for dsRNA synthesis; yellow frame, the position of lipoprotein N-terminal domain (LPD_N) domain; blue frame, unknown function motif 1943 (DUF1943) domain; greed frame, Von Willebrand factor type D domain (VWD); red frame, the site of the promoter or terminator.
