# Supplementary material for: Vitellogenin and Vitellogenin-Like Genes in the Brown Planthopper
Source: Front Physiol. 2019 Sep 18;10:1181. doi: 10.3389/fphys.2019.01181 (PMC6759490; doi:10.3389/fphys.2019.01181)
Supplement: Figure S2 — Nucleotide and deduced amino acid sequence of the NlVg-like1 coding region. Blue, the location of primers designed for RT-qPCR; red, the location of primers designed for dsRNA synthesis; yellow frame, the position of lipoprotein N-terminal domain (LPD_N) domain; blue frame, unknown function motif 1943 (DUF1943) domain; greed frame, Von Willebrand factor type D domain (VWD); red frame, the site of the promoter or terminator. [file Data_Sheet_2.PDF]

1 ATGGATCGATTTGTGGCGATAATTGCTCTAGGTTTCCTAATTGTTGGAACGGCATCACAAGAGGCGGAATACTTGTTCTTGCCCGAGCATGAATCAGTGTACGCGTGGAATGTAACCCTCACAACAGGGGCAGCTCTGCCCCAATCCGTCAGTTTATCGTGGAAT
1 M D R F V A I I A L G F L I V G T A S Q E A E Y L F L P E H E S V Y A W N V T L T T G A A L P Q S V S L S W N
166 TTCTCTGCAAATATCCATGTTCAACAGACAACTAGTAACACTACCATTTTCAAGATAAGTGATGTATTGACTTCGGAAAACAATGCAGTTGAAACACGGCCCATGCTGCAGCCTTTCAAGGCCACTTTTGAGAAGGGCCTACTTACAGCACTAATTACTGAGGAC
56 F S A N I H V Q Q T T S N T T I F K I S D V L T S E N N A V E T R P M L Q P F K A T F E K G L L T A L I T E D
331 TCCGACAAAAAGTGGTCTGTCAATATTAAAAGAGCCCTGGTCACTCTTCTACAATTGGACTTGGCTAGTATGAGTCAGGTGGCCTTTCTGTCGTCTGAGAGTGGCATGTATGGCACTTGTGTGACACATTACATTGTGACGAATGGAGATAAAAAGGTGACAGTG
111 S D K K W S V N I K R A L V T L L Q L D L A S M S Q V A F L S S E S G M Y G T C V T H Y I V T N G D K K V T V
496 CAGAAAATAGTTGACATGGACTCTTGTGCGAGTGGCACCAACCAAGATTCTCATCAATGGAGTAGTGCTCCGAATTTCATGTGTCCGAATAAATATCAAAAAGAGGTGATTAGTCACAGTGAAAGGACGTATATTCTGGATCCAAGTGCCAAGAGTCTAATCCAG
166 Q K I V D M D S C A S G T N Q D S H Q W S S A P N F M C P N K Y Q K E V I S H S E R T Y I L D P S A K S L I Q
661 TCAATCTCAAGCAAAGGAAAAATTTCACTACAGCCCTACCAATCTCAGGCTGAAGCTCATTACTCCTTGATAAGCCAGTCGTTGAATCTTGTGAGAGTGGACAAAATCGATAAGCTGTATGAAGTGGTTGAGAGCGCCGAAGAAACGAATCTGCTGTGGCAACCA
221 S I S S K G K I S L Q P Y Q S Q A E A H Y S L I S Q S L N L V R V D K I D K L Y E V V E S A E E T N L L W Q P
826 ATCAACCTGGATCCCACCTACGGAAAGCCTCCTACCAATAAAAGTGACGTCGTTTTGAAAGAGATCCAAAACATGCTTGGCGAGGTGAGTGACAGTCTGTCATGGTGGAAGGTGAAGGCGCAAGGCCTGCACAATGAGACGCTGTTCCGGCTGGTCGACCTCATG
276 I N L D P T Y G K P P T N K S D V V L K E I Q N M L G E V S D S L S W W K V K A Q G L H N E T L F R L V D L M
991 TGGTGGCTCGAACTCGACGACTGGCGCACGCTCTACAACACTGTCACCCTTGGCACCAGCTATCGACAGGAAACCATACAGCAAATCTTCTGGGAAGTAGTGCCTGAGGTGGGATCTGCGGCGTCGACAGTGTTCGTGAAGGATCTGGTACGCTCCAGCCAGGTC
331 W W L E L D D W R T L Y N T V T L G T S Y R Q E T I Q Q I F W E V V P E V G S A A S T V F V K D L V R S S Q V
1156 AAAGGCCTCAACGCCGTCTTCCTGCTTGGACGTCTCCCCTTCCGACTACGCAATCCTACTGAGCAACTTCTGTATCAATGCGAGGATCTTCTGCGACTGGGAGATGTTGGAGAAGAAGTGAAAAATTCGGCAATCTTATCCTTTGCAACGATGATCTATAAAACG
386 K G L N A V F L L G R L P F R L R N P T E Q L L Y Q C E D L L R L G D V G E E V K N S A I L S F A T M I Y K T
1321 TGTGCTGTTAGCTGCAAACCTGATACTGTGGACAGGTACACTAAATTGTTCCTGGACAGGTTCAAAGAAAGTACACTACATGGAGACAGAATGCTGTACCTACAAGCGCTGAGTAACATGGGATTGCCTCAGATACTCGATTACCTGTCGCCNGTCATTGCTGGC
441 C A V S C K P D T V D R Y T K L F L D R F K E S T L H G D R M L Y L Q A L S N M G L P Q I L D Y L S P V I A G
1486 AAAACCTCCAACGACCGGCATTTGCGATTCCTCGCCACTTGGGCTGTTATGCCAGCAGTCTTCACCGACCCTGCTAAGGTTCATGAGATATTCTGGCCACTGCTTGTGAACAGAACGGAGTCGCTAGAGATGCGAGTGGCGTCGCTGACTCTGCTGATTCTGGCC
496 K T S N D R H L R F L A T W A V M P A V F T D P A K V H E I F W P L L V N R T E S L E M R V A S L T L L I L A
1651 AAGCCGACGCCGGCCGGGCTGATCAGCCTGTTCTGGTACATGGCCAACGAGCCCAGTCAGCAGCTCTACCAGTTCTACTACACTACCATACAGTCACTCACCCAGACTACCTATCCTTGTTACACGCAACTTGGCGTGGTGGCTTCTCAGCTGGCCCGGTTCGTG
551 K P T P A G L I S L F W Y M A N E P S Q Q L Y Q F Y Y T T I Q S L T Q T T Y P C Y T Q L G V V A S Q L A R F V
1816 CAGCCTCGCTCGCACAGCTGGGCGACAGGCAACTACATCCTGGACTACGAGGAGCCCGACCGCGGATATGATGGCCTGCTGCAGATGCTGCTCATCGGCAGCGAGAGGACCGGCCTACCCAATGTCATCATGGTGGTCGCAGAGCAGCATGCGCTCGGACTCACC
606 Q P R S H S W A T G N Y I L D Y E E P D R G Y D G L L Q M L L I G S E R T G L P N V I M V V A E Q H A L G L T
1981 ACCGAACATGCTGTGTACCTGAAACTGGAAGGACTGAGCGAGGCCATGAAACACGTGGTGGCCAGTAAGCCGGTGAAGAGTGTGCAGAAAGTGCTGGAAGTGCTGCAGAACATGAAAGCACCCGACAGGGATGGCGAGAAAATCCATTTCGAGTTCATTCTGAAG
661 T E H A V Y L K L E G L S E A M K H V V A S K P V K S V Q K V L E V L Q N M K A P D R D G E K I H F E F I L K
2146 GCTGATGGTCGAACGGTCATGACTTATTTCATGAATGAAACCAACTTTCATAATTTGACTGCCGCTGCAAAGAAGTTGAGTTCGCTATACTTTGAATTCAGTGTGAACTACCAGGGACTGACATTCCCATTGGCCCTGCTGAAGCACCAGCCGACCGACTTCGGG
716 A D G R T V M T Y F M N E T N F H N L T A A A K K L S S L Y F E F S V N Y Q G L T F P L A L L K H Q P T D F G
2311 GTAACGGCTCTGTCGCAGGTGCGCTTCTCGTCGTTCATGTCGGCCAGGGGTCGCGTCTGTCAGGATGAGGAGGGAGCGGCCAGAAACGCCGAGTTGGATTTGAGATATTCGTGGAATGGCATCACCAGTCTGCGAGTTTTCAGTCCACTGAGCAACACTTGGTAC
771 V T A L S Q V R F S S F M S A R G R V C Q D E E G A A R N A E L D L R Y S W N G I T S L R V F S P L S N T W Y
2476 GGAGCTGACAGGAGCAGAAGTATTCATATTCGAGTCCCATTTGCCACCCACATCACGCTCAACTTTGCCAAATCGCACCTCAAGATCGTCGCTGTTAAGCATAGAGATTTCATGGCCGGTTCACAGATGGGAGGTGTATGGCATTCTGTTACAAAACTTATTTCG
826 G A D R S R S I H I R V P F A T H I T L N F A K S H L K I V A V K H R D F M A G S Q M G G V W H S V T K L I S
2641 GGAAGTACAACGATAGATCCTCCTCACAATATAACTGATGAATGGACAATGGACTCTGAAGATTTAGGAGCTCGGCTTGGCGCTAGTGTGTTCGACTGCCCTGGACAAACATTTGGAAATGCATTGCATTTACTGAAGAGAGCTTTCTTGGCAAAAAATAAAAAC
881 G S T T I D P P H N I T D E W T M D S E D L G A R L G A S V F D C P G Q T F G N A L H L L K R A F L A K N K N
2806 TATCATATGTTGCCTGGTGGAGTTGCCCTTCTTGGTCTGTTCTCCCTCAAGGATCAGTTGGCATTCCAGCCACCAGGGGGAGCTTGTGGTGTTCTTCTGTCTTTTGTGCCGCTCTTGACTCAGGTGGAGCCAGTGCCGCTATTAGAGAAGAACAAGCTGAGTCTG
936 Y H M L P G G V A L L G L F S L K D Q L A F Q P P G G A C G V L L S F V P L L T Q V E P V P L L E K N K L S L
2971 TCGATGACGCGTCGCGACGGCCTGCTGTGGGAGATCCAGGCGGCTATGAAGCAGCTTCACGACGGCAACGGTGAGGCTGCATTTAAGCTGTACCACGCGCCCAGCGTCTCAGTGTCGTCCGGCGGCTTCTGGCGCGTCATACAGCTGGAGGGCGCTTTCATCATT
991 S M T R R D G L L W E I Q A A M K Q L H D G N G E A A F K L Y H A P S V S V S S G G F W R V I Q L E G A F I I
3136 CCCTCGCGCAAGTCGGGTGTCTTTCATCCGCCCGCTCCTATCACTGGTTACACGTTTGTGTCATGGGGCGACGCGCAGCCAAGCAATTCGGACAAGTTGTCAGTGCTGGACGTGAAGGTGGTGCCTGGGAACAACATCAGCCAGCAGTCCCTCTGCTCCGACTTC
1046 P S R K S G V F H P P A P I T G Y T F V S W G D A Q P S N S D K L S V L D V K V V P G N N I S Q Q S L C S D F
3301 AACCCCATCTGTCTGCAGGCGTTGACCGATCTCGCCGCCAGGCAAACTGCCAATGTCCAGTACCTCAATCTTCCTGTTTGGTTCAAAACAGCTGCACATGCTGTCTTTCCTGAACATTTTCAAACTGAGAGTACGTCAGCCACATTCACATTCAATAGTCCTGTA
1101 N P I C L Q A L T D L A A R Q T A N V Q Y L N L P V W F K T A A H A V F P E H F Q T E S T S A T F T F N S P V
3466 CCGTTCCCCTGGAATACGAAAGGTCTTTGTGCAGTGAGCAAGAATGTCATCTTGACATTTGACAATGCAACTCTTGCTTCAACTATGCCTGAAGAATACACGCTGGCCGTTGCTGACTGCTCTGCTCACAAGCAATTTGCAATTCTCACCAAAAAAGTACCTGAA
1156 P F P W N T K G L C A V S K N V I L T F D N A T L A S T M P E E Y T L A V A D C S A H K Q F A I L T K K V P E
3631 AGCGACACTTTGGTGGTGAAACTGTTATTTGGCACAAAAGACGTTGAAATAATACCGACATTGAGTGGAAAACTGAAATTTGTACTGAATGGAATACCTCTTTCGGATAATGAGTTTACTCAAAACTTCGGAAACTCTTCAACCACAGATTTGCTAATTTTAGTG
1211 S D T L V V K L L F G T K D V E I I P T L S G K L K F V L N G I P L S D N E F T Q N F G N S S T T D L L I L V
3796 AAAGACAATGGCATGACTGAAGCTGAGTTGGAGAATGGAGTAGTTCTGCAGCACTATAACTCTACAATAGTTGTTCTCGTTCCTGGACTGTTCCGTAGTTTCACCTGTGGATTATGTGGAGACTTCAATAGTGACACTAACAACGACCCCATCCACATGTTCAAC
1266 K D N G M T E A E L E N G V V L Q H Y N S T I V V L V P G L F R S F T C G L C G D F N S D T N N D P I H M F N
3961 TACATCAAGTGA
1321 Y I K *

Figure S2. Nucleotide and deduced amino acid sequence of the *Nl*Vg-like1 coding region. Blue, the location of primers designed for RT-qPCR; red, the location of primers designed for dsRNA synthesis; yellow frame, the position of lipoprotein N-terminal domain (LPD_N) domain; blue frame, unknown function motif 1943 (DUF1943) domain; greed frame, Von Willebrand factor type D domain (VWD); red frame, the site of the promoter or terminator.
